# Multilevel fine-scale diversity challenges the ‘cryptic species’ concept

**DOI:** 10.1038/s41598-019-42297-5

**Published:** 2019-05-01

**Authors:** Tatiana Korshunova, Bernard Picton, Giulia Furfaro, Paolo Mariottini, Miquel Pontes, Jakov Prkić, Karin Fletcher, Klas Malmberg, Kennet Lundin, Alexander Martynov

**Affiliations:** 1 Koltzov Institute of Developmental Biology RAS, 26 Vavilova Str., 119334 Moscow, Russia; 2 Zoological Museum, Moscow State University, Bolshaya Nikitskaya Str. 6, 125009 Moscow, Russia; 3 National Museums Northern Ireland, Holywood, Northern Ireland BT18 0EU United Kingdom; 4 Queen’s University, Belfast, Northern Ireland United Kingdom; 5 Department of Science, University of “Roma Tre”, Viale G. Marconi 446, I-00146 Rome, Italy; 6 VIMAR - Vida Marina, Barcelona, Spain; 7 Getaldiceva 11, C 21000 Split, Croatia; 8 Port Orchard, Washington, 98366 USA; 9 Aquatilis, Nostravägen 11, S-41743 Gothenburg, Sweden; 10 Gothenburg Natural History Museum, Box 7283, SE-40235 Gothenburg, Sweden; 11 Gothenburg Global Biodiversity Centre, Box 461, SE-40530 Gothenburg, Sweden

**Keywords:** Phylogenetics, Speciation, DNA sequencing, Zoology, Biodiversity

## Abstract

‘Cryptic’ species are an emerging biological problem that is broadly discussed in the present study. Recently, a cryptic species definition was suggested for those species which manifest low morphological, but considerable genetic, disparity. As a case study we present unique material from a charismatic group of nudibranch molluscs of the genus *Trinchesia* from European waters to reveal three new species and demonstrate that they show a dual nature: on one hand, they can be considered a ‘cryptic’ species complex due to their overall similarity, but on the other hand, stable morphological differences as well as molecular differences are demonstrated for every species in that complex. Thus, this species complex can equally be named ‘cryptic’, ‘pseudocryptic’ or ‘non-cryptic’. We also present evidence for an extremely rapid speciation rate in this species complex and link the species problem with epigenetics. Available metazoan-wide data, which are broadly discussed in the present study, show the unsuitability of a ‘cryptic’ species concept because the degree of crypticity represents a continuum when a finer multilevel morphological and molecular scale is applied to uncover more narrowly defined species making the ‘cryptic’ addition to ‘species’ redundant. Morphological and molecular methods should be applied in concordance to form a fine-scale multilevel taxonomic framework, and not necessarily implying only an *a posteriori* transformation of exclusively molecular-based ‘cryptic’ species into morphologically-defined ‘pseudocryptic’ ones. Implications of the present study have importance for many fields, including conservation biology and fine-scale biodiversity assessments.

## Introduction

The ‘cryptic species’ concept is widely used in modern biodiversity studies^1^ and implies that there are morphologically indistinguishable species that can be recognized only by molecular data^2,3^. The term ‘cryptic species’ became popular relatively recently^4,5^ and has supplanted the term ‘sibling species’ which was commonly used in previous cases with difficult-to-distinguish species^6^. Despite the fact that the ‘cryptic species’ concept has received considerable attention and is now used in various applications, it is intrinsically contradictory and might also be confused with other uses of the word ‘cryptic’. For example, the exact same ‘cryptic species’ term is used in ecology to denote that some species are very well camouflaged on some substrates^7^.

Recently, the difficulty of delineating the ‘cryptic species’ concept from the basic biological species definition was highlighted, and it was suggested that this concept should be used with care and only as a temporary formalization for taxonomic complexes for which a robust morphological framework is not yet established^8^. In a further discussion, Struck *et al*.^9^ rather disagreed and attempted to build a definition of ‘cryptic’ species on a lower degree of phenotypic (morphological) disparity than non-cryptic relatives. However, to define an exact degree of morphological disparity is very difficult, if it is possible at all, so Heethoff^10^ further concluded that the current ‘cryptic species’ concept represents either conceptual or terminological chaos. Additional complications arise from the most recent practice in different animal groups such as crustaceans^11^, insects^12^ or echinoderms^13^ when the term ‘cryptic’ species is used for morphologically diagnosable species.

The ‘cryptic’ species problem is not merely a theoretical problem. The strong trend to distinguish ‘cryptic’ and ‘non-cryptic’ species, and the explosive growth of exploration and description of ‘cryptic’ diversity, without a clearly defined terminological ground, is already resulting in recent outstanding controversies in important fields such as conservation biology - particularly regarding suggestions for special regulations of species names, that will affect the core of taxonomy^14,15^. The term ‘cryptic species’ is also commonly vaguely applied, making its definition uncertain^10^, which prompts the necessity of its revision. The present case is of special importance since it links a taxonomic problem in a particular organism group with the most general biological problem of the species concept, using broad-scope material on marine animals. Here, we show that a very colourful species of nudibranch mollusc *Trinchesia caerulea* (Montagu, 1804), which was originally described from the UK, but common throughout European waters (including Norway, Sweden and the Mediterranean region), is revealed to actually be a complex of four species (three of which are new). According to the present study, these species are robustly distinguished by their molecular data and were never taxonomically separated previously and thus fit within the ‘cryptic species’ concept and its updated definition including “low morphological disparity^9^”. However, the apparent cryptic morphology for that complex had previously been noted and questioned^16,17^ and in the present study those morphological distinctions have been supported by novel molecular data. Thus, these ‘cryptic’ species can be distinguished morphologically (and hence can be defined as ‘pseudo-cryptic’ species) not only *a posteriori* after molecular study, as was commonly suggested and has become a notable modern tendency^18,19^, but by using multilevel morphological information as a primary source. This adds a new perspective to the understanding of the core biological species problem, which should not just be to adhere to a putative cryptic notion, but methods should rather follow the idea “from cryptic to obvious species” with the rapid progress of various molecular analyses and aids to morphological differentiation^20,21^ in order to reveal an immense hidden multilevel biological diversity.

## Materials and Methods

Material for this study was obtained by scuba diving at widely separated locations across Europe: from the United Kingdom, Ireland, Norway, Sweden, Spain, Italy, France, Croatia and Russia. The specimens were deposited in the Zoological Museum of Lomonosov Moscow State University (ZMMU), in the Gothenburg Natural History Museum (GNM), National Museums Northern Ireland (BELUM.Mn), and the Department of Science of the Roma Tre University (RM3). Integration of molecular and morphological data as well as phylogenetic and biogeographical patterns were used. The external and internal morphology of the 28 specimens was studied using digital cameras, under a stereomicroscope and scanning electron microscope. For molecular analysis 17 specimens were successfully sequenced for the mitochondrial genes cytochrome c oxidase subunit I (COI), 16S rRNA, and the nuclear genes Histone 3 (H3). The DNA extraction procedure, PCR amplification options, and sequence gathering are described in detail in previous studies^8,22–25^. Additional molecular data for 13 specimens of nudibranchs were obtained from GenBank (see supplementary information Table S1). Outgroup selection was based on previous studies^23,25–27^. Two different phylogenetic methods, Bayesian Inference (BI) and Maximum Likelihood (ML), were used to infer evolutionary relationships. To evaluate the genetic distribution of the different haplotypes, a haplotype network for the COI molecular data was reconstructed using Population Analysis with Reticulate Trees (PopART, http://popart.otago.ac.nz). Also, the minimum uncorrected *p*-distances between all the sequences as well as maximum intra- and minimum intergroup genetic distances were examined. Automatic Barcode Gap Discovery (ABGD)^28^ was used to define species. See supplementary information Text for methods in detail.

## Results

### Molecular phylogenetic relationships and morphological data of a nudibranch species complex

**Figure 1:**
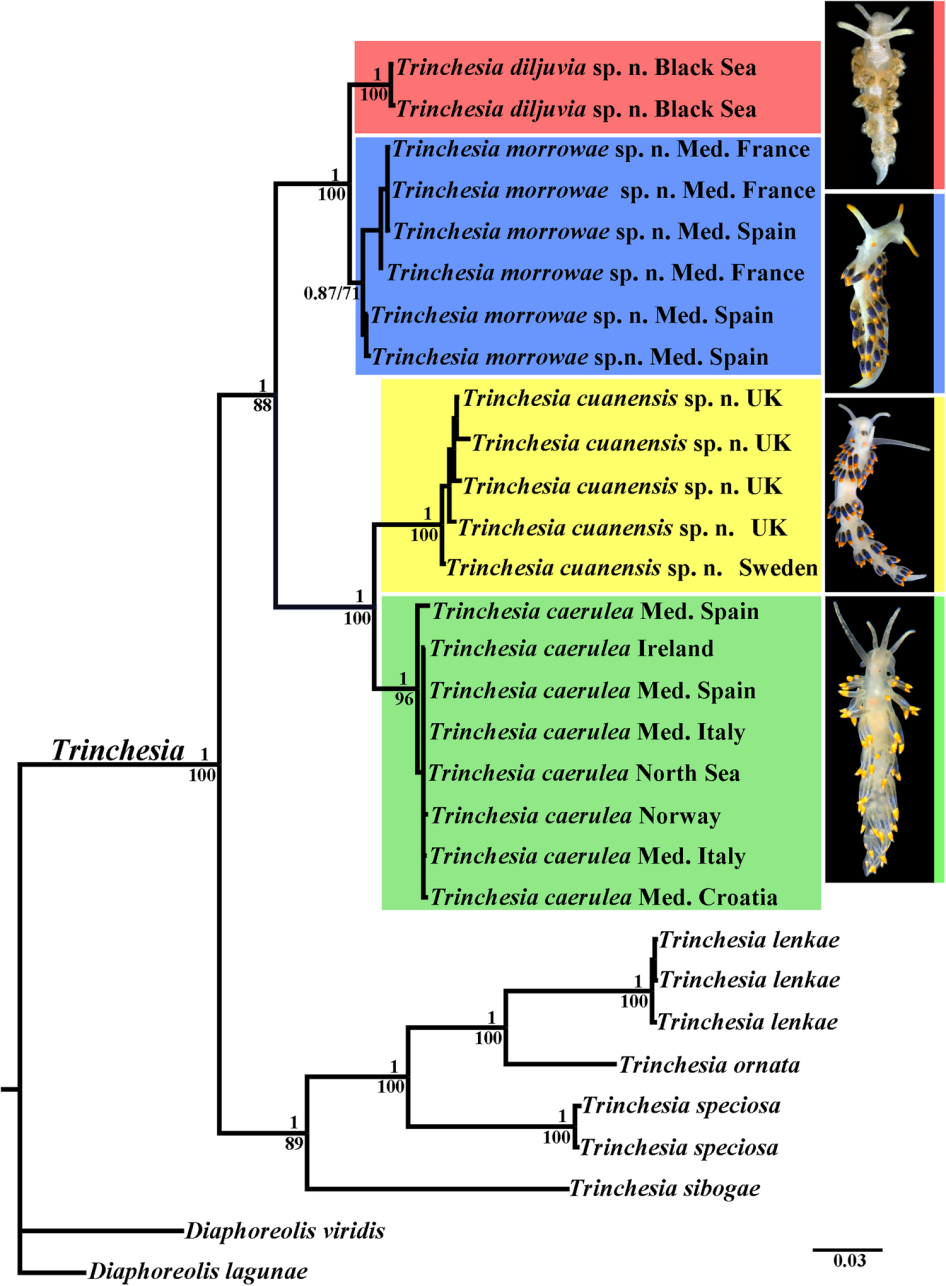
Phylogenetic relationships of nudibranchs *Trinchesia* based on COI + 16S + H3 concatenated dataset inferred by Bayesian Inference (BI). Numbers above branches represent posterior probabilities from Bayesian Inference. Numbers below branches indicate bootstrap values for Maximum Likelihood. A comparison of the external morphology is indicated by arrows. Abbreviations: Med. – The Mediterranean Sea; UK – United Kingdom. Photos by B.P. and T.K.

**Figure 2:**
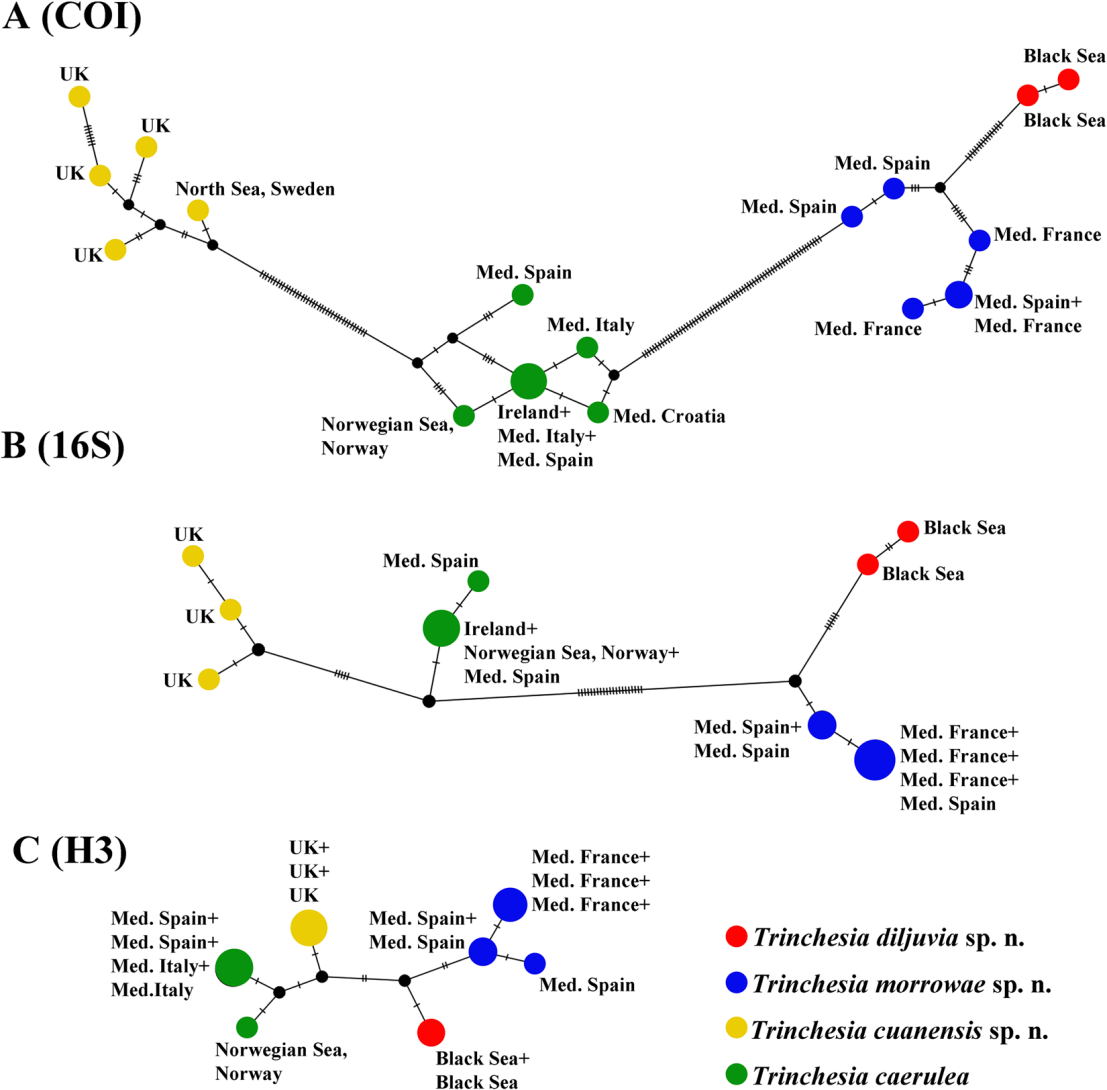
The haplotype network based on (**A**) cytochrome c oxidase subunit I (COI), (**B**) 16S rRNA, and **(C)** –Histone 3 (H3) molecular data showing genetic mutations occurring within *Trinchesia caerulea* complex species.

**Figure 3:**
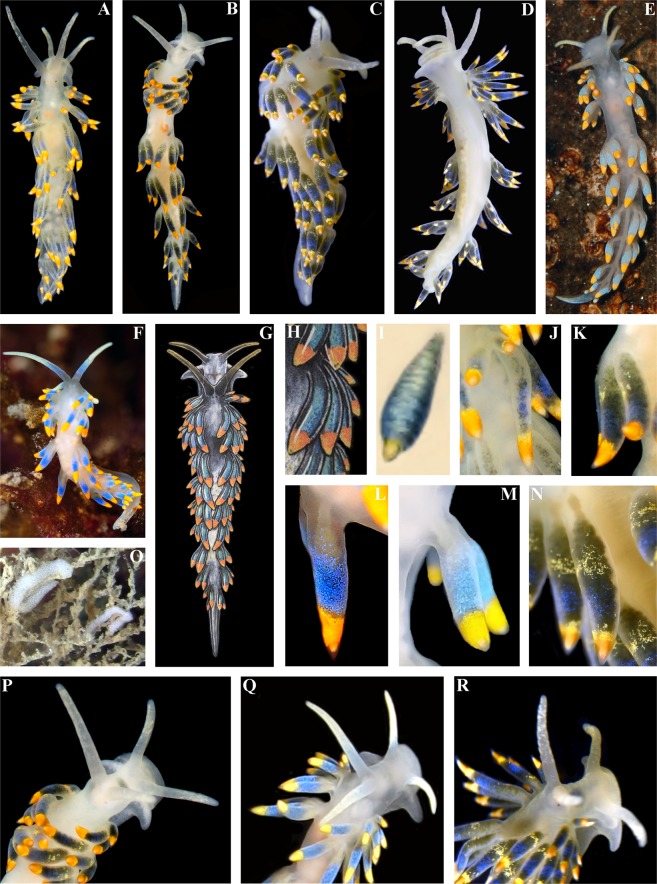
*Trinchesia caerulea* (Montagu, 1804). External views of living specimens and comparison with data from Thompson & Brown (1984). (**A**) Neotype, Ireland, Mayo, Killary Harbour, Rusheen Point (ZMMU Op-646). (**B**) Specimen from Wales, Pembrokeshire, Martin’s Haven (BELUM.Mn2018.1). (**C**) Specimen from Norway, Gulen Dive Resort (ZMMU Op-622). (**D**) Same specimen, ventral view. (**E**) Specimen from Italy. (**F**) Specimen from Spain, Girona, L’Estartit (ZMMU Op-648). (**G**) Specimen from Wales, Pembrokeshire, Skomer Is., depicted in Thompson & Brown (1984: pl 30, c). (**H**) Same, details of cerata. (**I**) Details of ceras from original description of *T*. *caerulea* in Montagu (1804), specimen from UK (Devon). (**J**). Details of cerata of specimen from Ireland. (**K**) Details of cerata of specimen from Wales. (**L**) Details of ceras of specimen from Spain. (**M**) Details of cerata of specimen from Italy. (**N**) Details of cerata of specimen from Norway. (**O**) Egg mass from Italy. (**P**) Details of anterior part of specimen from Wales. (**Q**) Details of anterior part of specimen from Italy. (**R**) Details of anterior part of specimen from Norway. Photographs: Bernard Picton, (a), (b), (d), (e), (j), (k), (p); Tatiana Korshunova: (c), (n), (r); Miquel Pontes: (f), (l), (q); Giulia Furfaro: (o). Reproduction of figures from Thompson & Brown (1984) with permission of Gregory Brown, original artist and copyright holder of the images.

**Figure 4:**
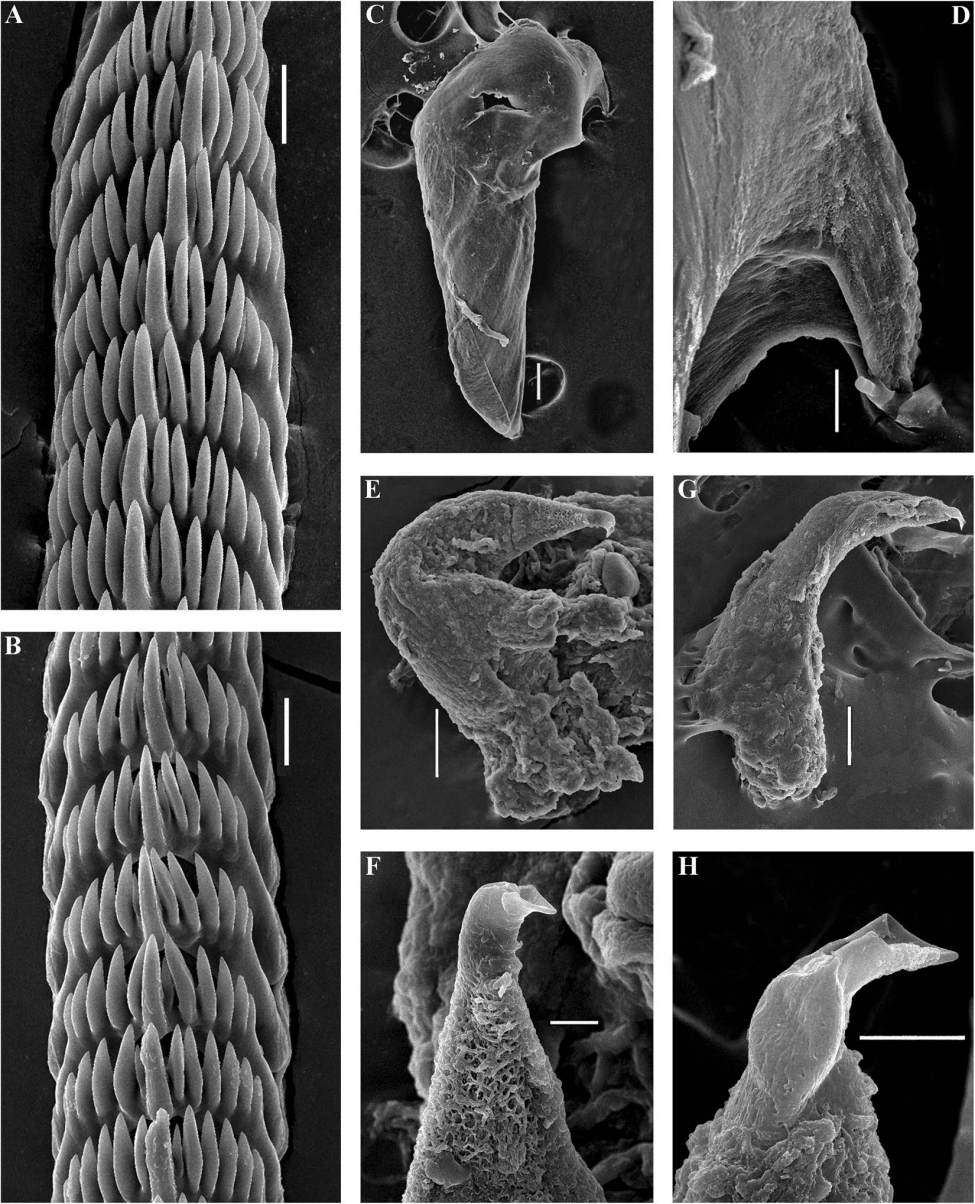
*Trinchesia caerulea* (Montagu, 1804). Internal morphology, scanning electron microscopy. (**A**) Posterior part of radula of specimen from Spain, L’Estartit, Girona (ZMMU Op-648). (**B**) Posterior part of radula of specimen from Norway, Gulen Dive Resort (ZMMU Op-622). (**C**) Jaw of specimen from Norway (Gulen). (**D**) Details of masticatory processes of jaws, same specimen. (**E**) Copulative organ with stylet of specimen from Norway. (**F**) Stylet, details, same specimen. (**G**) Copulative organ with stylet of specimen from Spain. (**H**) Stylet details, same specimen. Scale bars: a, b, d −20 μm, c −100 μm, e, g −50 μm, f, h −10 μm. SEM micrographs: Alexander Martynov.

**Figure 5:**
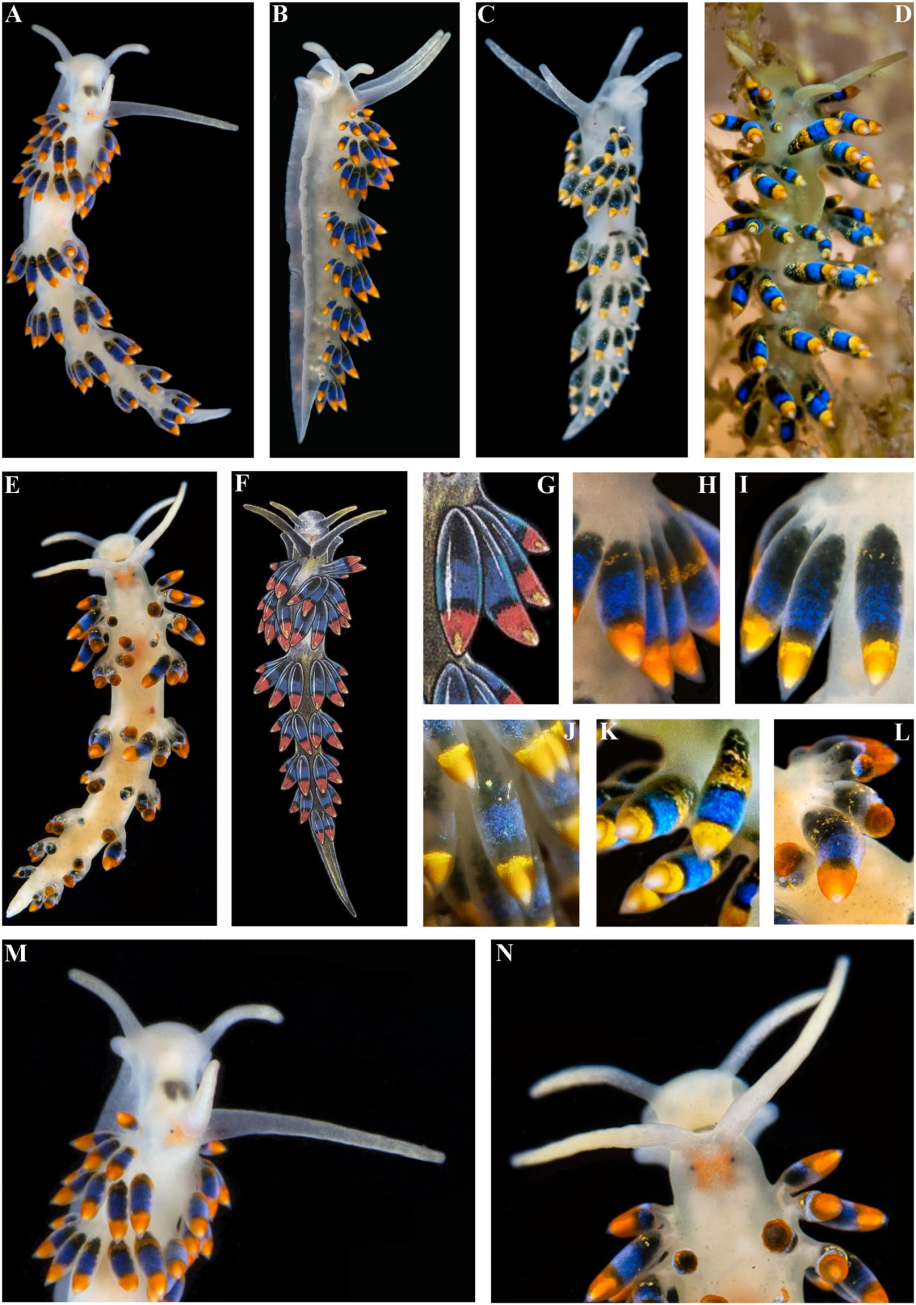
*Trinchesia cuanensis* sp. n. External views of living specimens and comparison with morphs included by Thompson & Brown (1984) as a single species “*Trinchesia caerulea*”. (**A**). Holotype, Down, Strangford Lough, Northern Ireland, United Kingdom (ZMMU Op-650), dorsal view. (**B**) Same specimen, left lateral view. (**C**) Paratype from Bohuslän, Smögen, Sweden (GNM-9243), right lateral view. (**D**) Paratype from Antrim, Portrush, United Kingdom, dorsal view (BELUM.Mn.2018.2). (**E**) Paratype from Down, Strangford Lough, dorsal view (ZMMU Op-655). (**F**) Specimen from Isle of Man, Port Erin, depicted in Thompson & Brown (1984: pl 30, c), dorsal view. (**G**) Same, details of cerata. (**H**). Details of cerata of the holotype. (**I**) Details of cerata, of paratype from Down, Strangford Lough. (**J**) Details of cerata of paratype from Sweden. (**K**) Details of cerata, of paratype from Antrim, Portrush. (**L**) Details of cerata, of paratype from Strangford Lough. (**M**) Details of anterior part of holotype from Strangford Lough. (**N**) Details of anterior part of paratype from Strangford Lough. Photographs: Bernard Picton, (a), (b), (d), (e), (h), (i), (k), (l), (m), (n); Klas Malmberg, (c), (j). Reproduction of figures from Thompson & Brown (1984) with permission Gregory Brown, original artist and copyright holder of the images.

**Figure 6:**
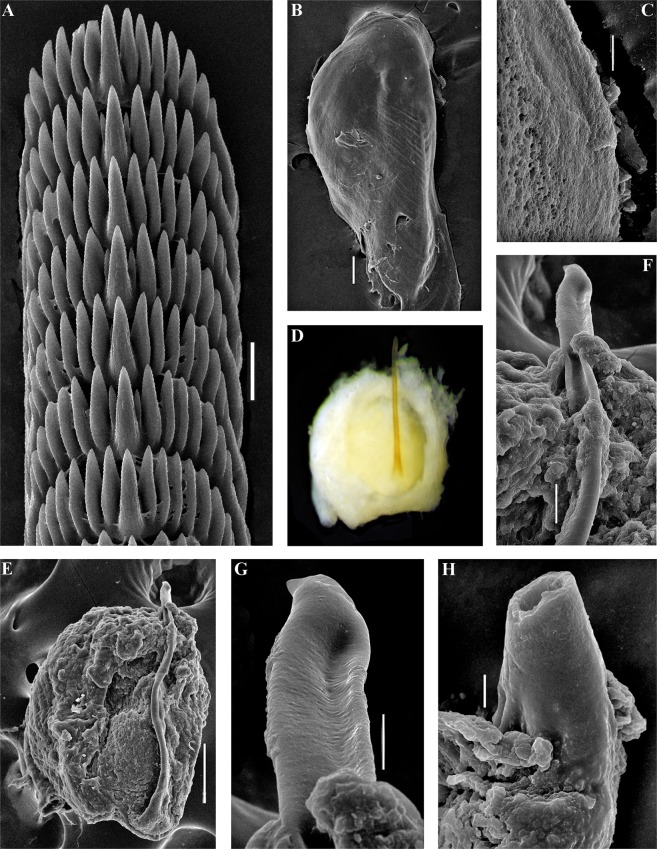
*Trinchesia cuanensis* sp. n. Internal morphology, scanning and light electron microscopy. (**A**) Posterior part of radula of holotype from Northern Ireland (ZMMU Op-650). (**B**) Jaw, holotype. (**C**) Details of masticatory processes of jaws, holotype. (**D**) Copulative organ with stylet of paratype from Northern Ireland (GNM Gastropoda – 9054), light microscopy. (**E**) Same, scanning electron microscopy. (**F**) Details of stylet, same paratype. (**G**) Details of apical part of stylet, same specimen. (**H**) Cross section of basal part of stylet, showing channel inside, holotype. Scale bars: a, f −20 μm, b, e −100 μm, c, h, g −10 μm. SEM micrographs: Alexander Martynov.

**Figure 7:**
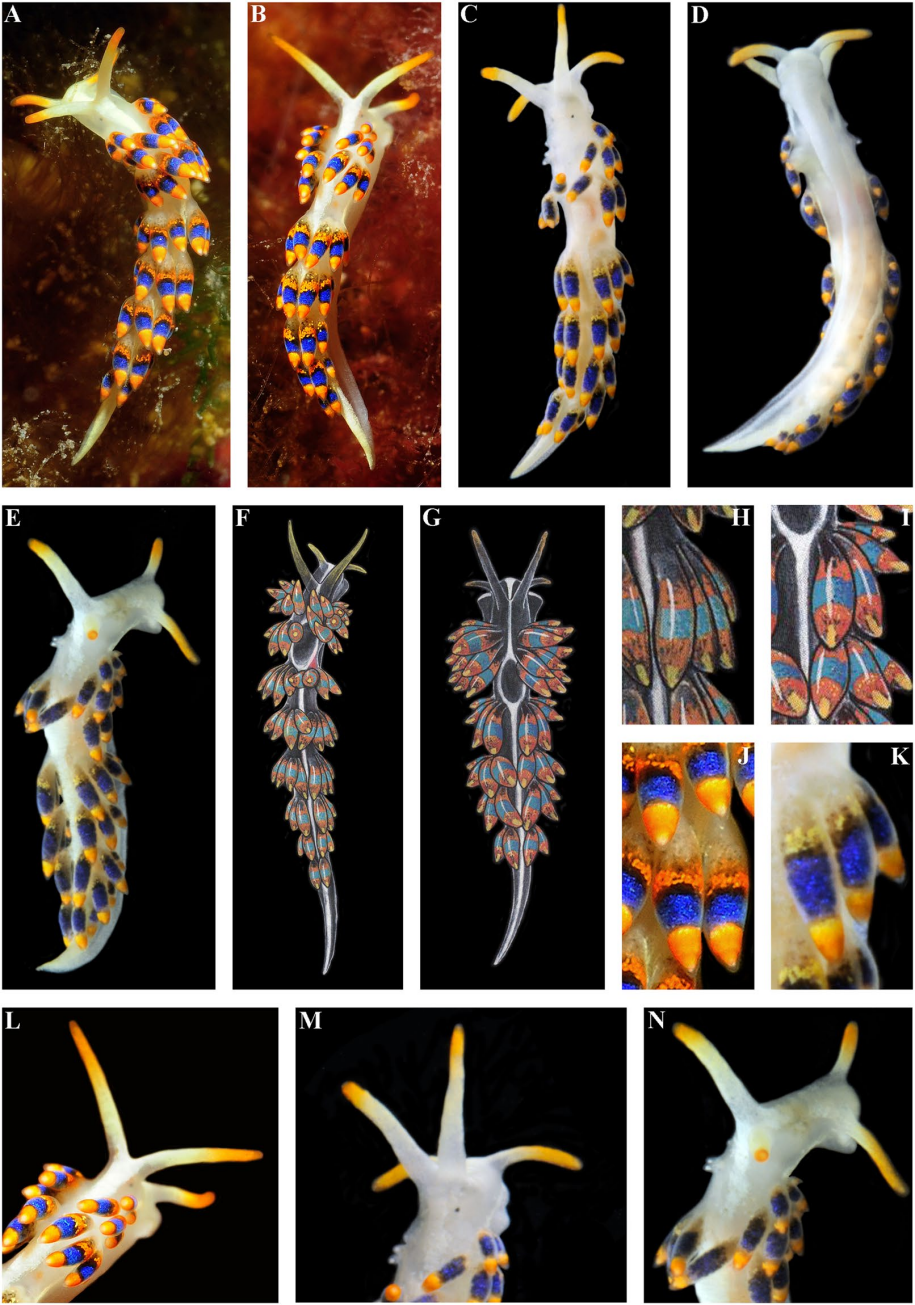
*Trinchesia morrowae* sp. n. External views of living specimens and comparison with morphs included in Thompson & Brown (1984) as the single species “*Trinchesia caerulea*”. (**A**) Holotype from Spain, Girona, L’Estartit, dorsal view (ZMMU Op-651). (**B**) Holotype from same location, dorso-lateral view (right side). (**C**) Paratype from France, Banyuls-sur-Mer (ZMMU Op-653), dorsal view. (**D**) Same paratype, ventral view. (**E**) Same paratype, dorso-lateral view (right side). (**F**). Specimen from UK (Lundy Island) depicted in Thompson & Brown (1984: pl 30, a), dorsal view. (**G**) Specimen from UK (Scilly Isles) depicted in Thompson & Brown (1984: pl 30, d), dorsal view. (**H**) Details of cerata of specimen from UK (Lundy Island) depicted in Thompson & Brown (1984: pl 30, a). (**I**) Details of cerata of specimen from UK (Scilly Isles) depicted in Thompson & Brown (1984: pl 30, d). (**J**) Details of cerata of holotype. (**K**) Details of cerata of specimen from France (ZMMU Op-653). (**L**) Details of anterior part of holotype. (**M**) Details of paratype from France. (**N**) Details of anterior part of the same paratype. Photographs: Miquel Pontes, (a), (b), (j); Tatiana Korshunova, (c), (d), (k), (m), (n). Reproduction of figures from Thompson & Brown (1984) with permission of Gregory Brown, original artist and copyright holder of the images.

**Figure 8:**
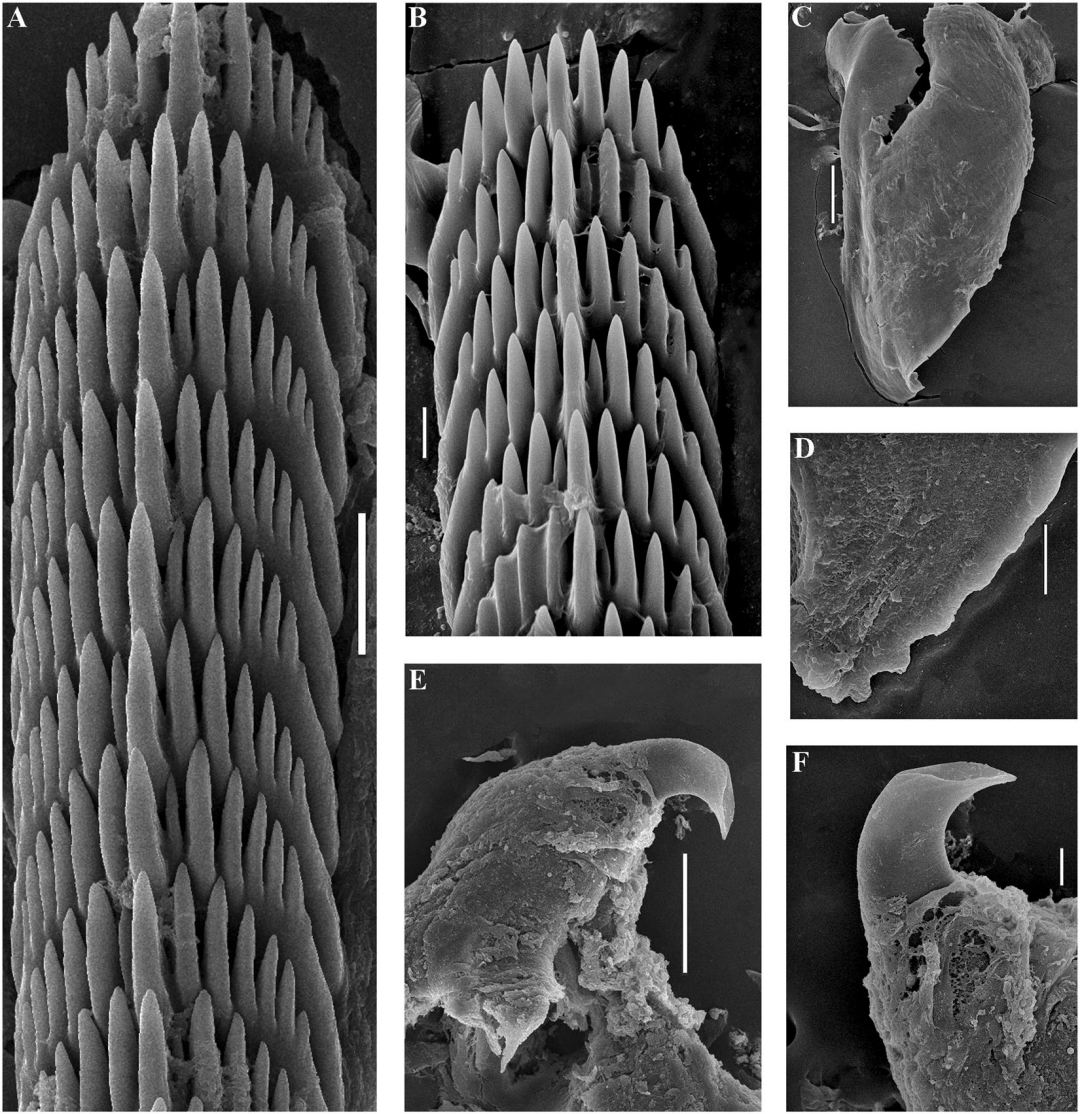
*Trinchesia morrowae* sp. n. Internal morphology, scanning electron microscopy. (**A**) Posterior part of radula of paratype from Spain, L’Estartit (ZMMU Op-652). (**B**) Posterior part of radula of paratype from France, Banyuls-sur-Mer. (**C**) Jaw of specimen from Spain (L’Estartit, Girona). (**D**) Details of masticatory processes of jaws, same specimen. (**E**) Copulative organ with stylet of specimen from France, Banyuls-sur-Mer (ZMMU Op-653). (**F**) Details of stylet, paratype. Scale bars: (a) −20 μm; (b), (d), (f) −10 μm; (c) −100 μm; (e) −50 μm. SEM micrographs: Alexander Martynov.

**Figure 9:**
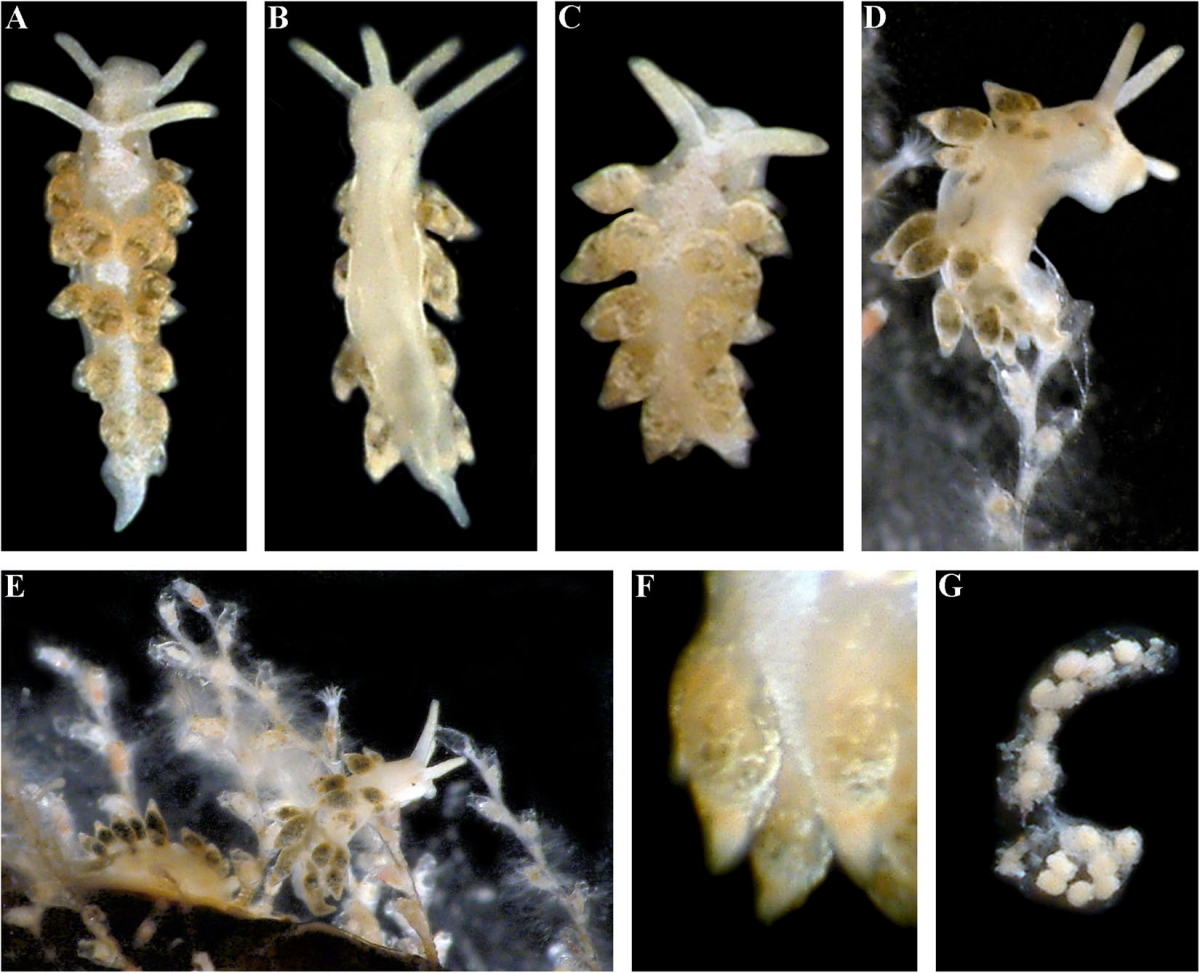
*Trinchesia diljuvia* sp. n. External views of living specimens. (**A**) Holotype, Black Sea, Yalta region (ZMMU Op-642) dorsal view; (**B**). Holotype, ventral view; (**C**). Paratype, Black Sea, Yalta region, dorsal view (ZMMU Op-645); (**D**). Paratype, Black Sea, Yalta region (ZMMU Op-643), lateral right view; (**E**). Living specimens from same location on food sources, hydroids; (**F**). Details cerata; (**G**). Egg mass from specimens from the same locality. Photographs: Tatiana Korshunova, Alexander Martynov.

**Figure 10:**
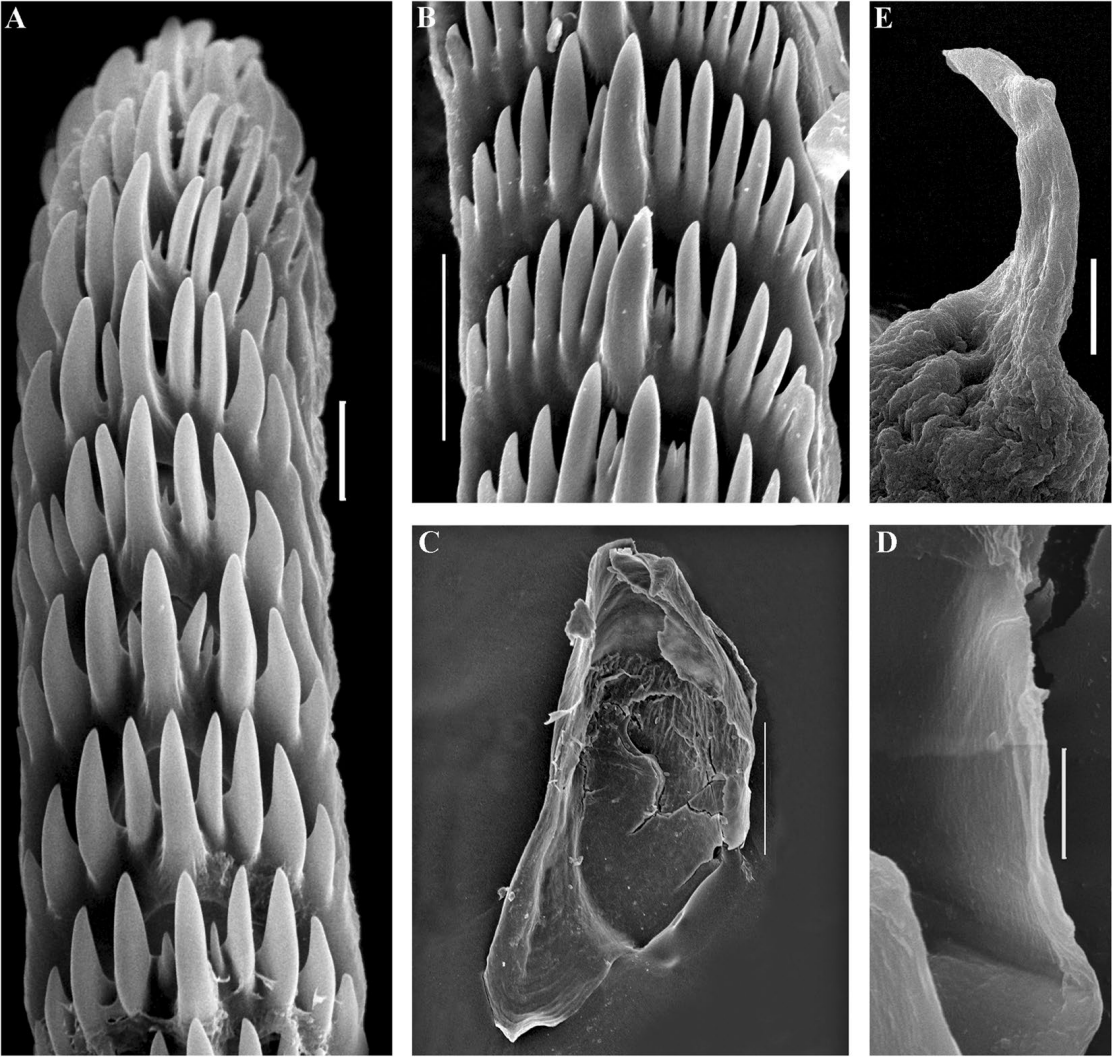
*Trinchesia diljuvia* sp. n. Internal morphology, scanning electron microscopy. (**A**) Posterior part of radula of paratype ZMMU Op-645; (**B**). Posterior part of radula (specimen ZMMU Op-643); (**C**). Jaw, paratype ZMMU Op-643; (**D**). Details of masticatory processes of jaws, same specimen; (**E**). Details of copulative organ with stylet, same specimen (ZMMU Op-645). Scale bars: (a), (d), (e) −10 μm; (b) −20 μm; (c) −120 μm; SEM micrographs: Alexander Martynov.

**Figure 11:**
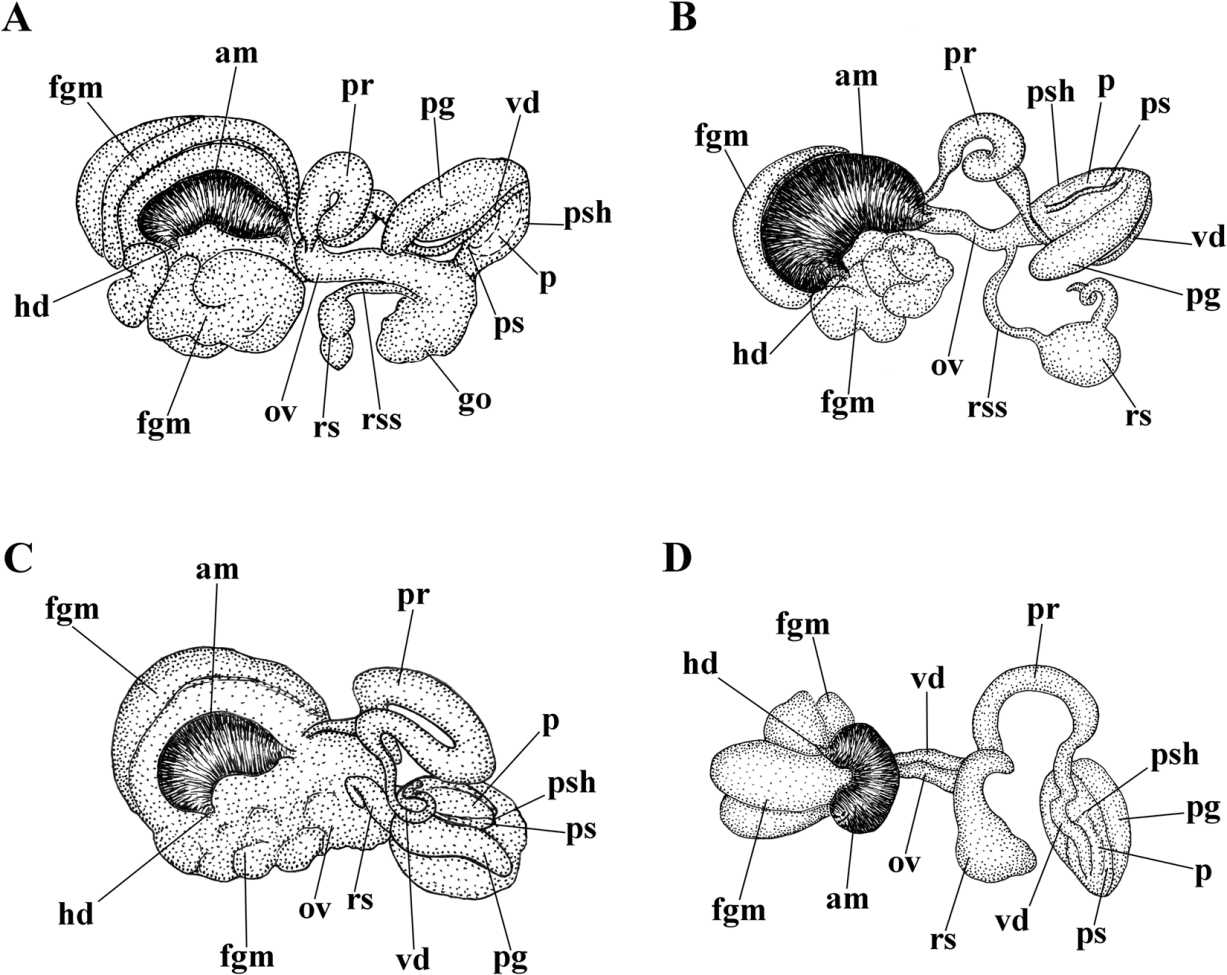
Reproductive systems, schemes. (**A**) *Trinchesia caerulea*. (**B**) *Trinchesia cuanensis* sp. n. (**C**) *Trinchesia morrowae* sp. n. (**D**). *Trinchesia diljuvia* sp. n. Schemes by Tatiana Korshunova. Abbreviations: am, ampulla; fgm, female gland mass; hd, hermaphroditic duct; ov, oviduct; p, penis; pg, “penial” (supplementary) gland; pr, prostate; ps, penial stylet; psh, penial sheath; rs, receptaculum seminis; rss, stalk of receptaulum seminis; vd, vas deferens.

Phylogenetic analysis was performed using 28 specimens of *Trinchesia*, including data for 21 *Trinchesia caerulea* species complex specimens, four other congener species of the genus *Trinchesia* and two outgroup species. All supposedly morphologically cryptic morphs of *Trinchesia caerulea* illustrated in Thompson & Brown (1984)^16^ were collected in European waters and examined. In addition, specimens collected in the Black Sea that are taxonomically close to *T*. *caerulea* were also included in the study. We apply the concept *‘Trinchesia caerulea* species complex’ here as the designation for all these studied specimens. The dataset consisted of 80 nucleotide sequences including mitochondrial COI and 16S, and the nuclear H3 genes. The SYM + G model was chosen for the concatenated dataset. Bayesian Inference (BI) and Maximum Likelihood (ML) analyses based on the combined dataset for the COI, 16S and H3 genes yielded similar results (Fig. 1). To define species, we use an integrative approach^25,29^ including phylogenetic tree topologies, ABGD analysis, pairwise distances and the haplotype network using PopART (Fig. 2, see also supplementary information Tables S1–S5). The results of the integrative study clearly identified four species in the *Trinchesia caerulea* species complex: *T*. *caerulea* (neotype is designated here, ZMMU Op-646), *T*. *cuanensis* sp. n. (holotype ZMMU Op-650, ZooBank registration: urn:lsid:zoobank.org:act: 77629514-DDB5-4757-86FE-78DDD7672563), *T*. *morrowae* sp. n. (holotype ZMMU Op-651, ZooBank registration: urn:lsid:zoobank.org:act: D7DB7FFB-F6B1-4A67-8DF4-A1D0F05963A7), and *T*. *diljuvia* sp. n. (holotype ZMMU Op-642, ZooBank registration: urn:lsid:zoobank.org:act: C9228E8B-DF2C-46A0-9E78-038ED6B87D7B). ZooBank registration for the paper is: urn:lsid:zoobank.org:act: 4B5F968F-69B6-4FB0-BB7B-43ABC4B09EBB. Information about the taxonomy of these four species can be found in the descriptions below, in the Figs 3–11 and in the supplementary information, Table S5.

The molecular phylogenetic analysis also supported the presence of these four species in the *Trinchesia caerulea* species complex: *T*. *caerulea* (PP = 1, BS = 96%), *T*. *cuanensis* sp. n. (PP = 1, BS = 100%),*T*. *morrowae* sp. n. (PP = 0.87, BS = 71%), and *T*. *diljuvia* sp. n. (PP = 1, BS = 100%). The maximum genetic distance values within the *T*. *caerulea* group are 1.25% for COI, 0.25% for 16S, and 0.66% for H3 markers. The maximum genetic distance values within the *T*. *cuanensis* sp. n. group are 1.88% for COI, 0.46% for 16S, and 0% for H3 markers. The maximum genetic distance values within the *T*. *morrowae* sp. n. group are 2.18% for COI, 0.23% for 16S, and 0.65% for H3 markers. The maximum genetic distance values within the *T*. *diljuvia* sp. n. group are 0.16% for COI, 0.46% for 16S, and 0% for H3 markers. Regarding the supposedly fast-evolving COI marker, minimum genetic distance values between the *T*. *caerulea* and the *T*. *cuanensis* sp. n., *T*. *morrowae* sp. n., and *T*. *diljuvia* sp. n. groups are 7.20%, 10.95% and 11.89% respectively. The minimal COI p-distance (2.98%) was found between *T*. *morrowae* sp. n. and *T*. *diljuvia* sp. n.; the maximal COI p-distance (12.99%) was found between *T*. *cuanensis* sp. n. and *T*. *diljuvia* sp. n. The minimal 16S p-distance (1.39%) was found between *T*. *caerulea* and *T*. *cuanensis* sp. n.; the maximal 16S p-distance (6.47%) was found between *T*. *caerulea* and *T*. *diljuvia* sp. n. The minimal nuclear H3 p-distance (0.98%) was found between *T*. *caerulea* and *T*. *cuanensis* sp. n. and between *T*. *morrowae* sp. n. and *T*. *diljuvia* sp. n.; the maximal nuclear H3 p-distance (1.63%) was found between *T*. *caerulea* and *T*. *morrowae* sp. n. and *T*. *caerulea* and *T*. *diljuvia* sp. n and between *T*. *cuanensis* sp. n. and *T*. *morrowae* sp. n. All minimum intergroup genetic distances for every marker are larger than the maximum intragroup distances. (see supplementary information, Tables S2–S4). Results obtained by PopART showed a network of haplotypes that clearly clustered into four groups coincident with *T*. *caerulea*, *T*. *cuanensis* sp. n., *T*. *morrowae* sp. n., and *T*. *diljuvia* sp. n. (Fig. 2). The ABGD analysis of the COI data set run with two different models with the initial approach revealed seven potential species of the genus *Trinchesia*: *T*. *caerulea*, *T*. *cuanensis* sp. n., *T*. *lenkae*, *T*. *ornata*, *T*. *sibogae*, *T*. *speciosa*, and *T*. *morrowae* sp. n. In the recursive approach, the additional species *T*. *diljuvia* sp. n. is recognized. The ABGD analysis of the 16S data set run with two different models with the initial approach revealed eight potential species: *T*. *diljuvia* sp. n., *T*. *caerulea*, *T*. *cuanensis* sp. n., *T*. *lenkae*, *T*. *morrowae* sp. n., *T*. *ornata*, *T*. *sibogae*, and *T*. *speciosa*. Detailed morphological investigation was carried out based on molecular phylogenetic delimitation.

**Figure 12:**
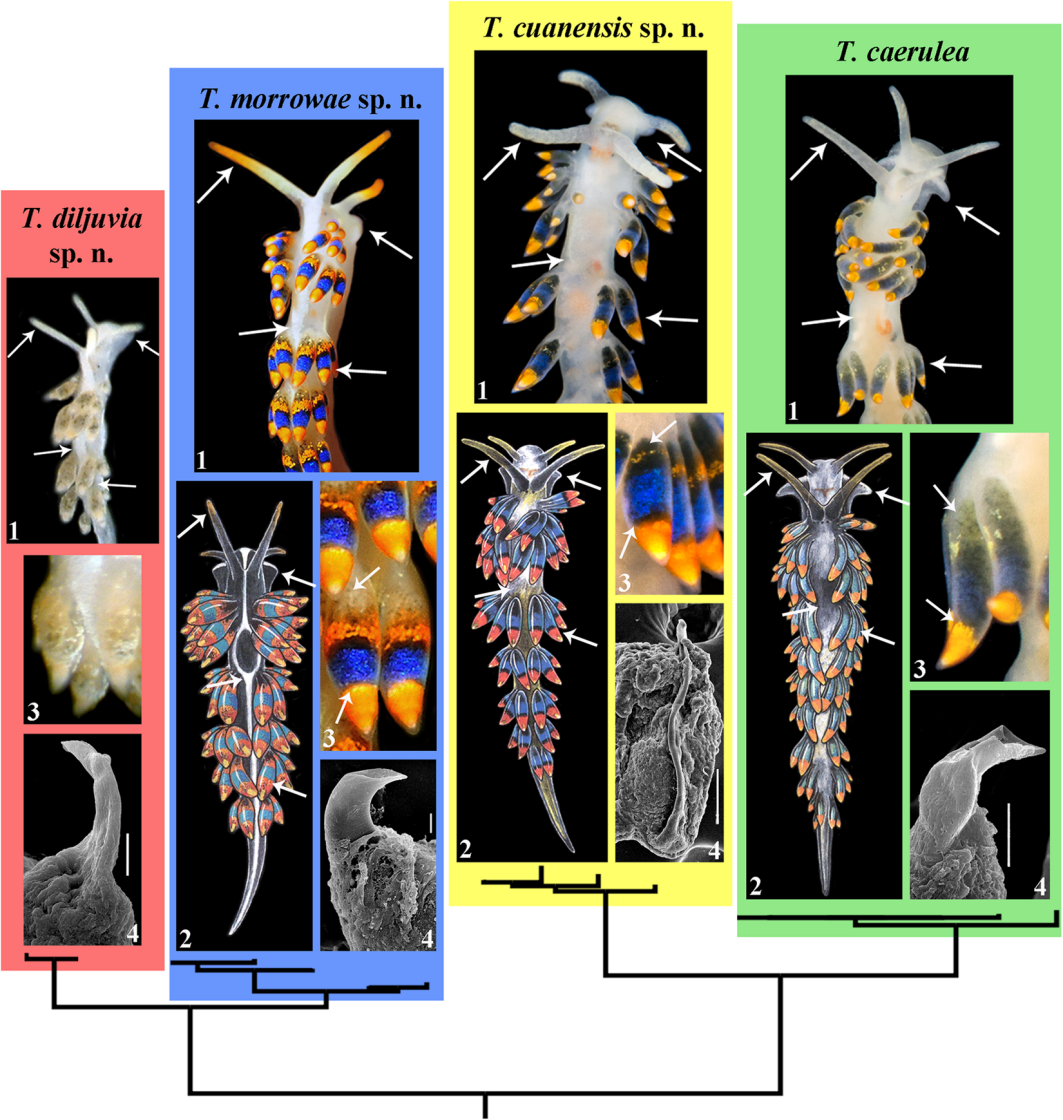
Multilevel integrative presentation of molecular (based on the molecular phylogenetic tree, Fig. 1) and morphological data for four species of the *Trinchesia caerulea* complex. For each species [*T*. *caerulea* (Montagu, 1804), *T*. *cuanensis* sp. n., *T*. *morrowae* sp. n., *T*. *diljuvia* sp. n.] external view of living specimen (1) (photos by B.P. and T.K.) is coupled with appropriate Fig. (2) of *T*. *caerulea* morphs from Thompson & Brown monograph (reproduced with permission of Gregory Brown, original artist and copyright holder of the images), without taxonomic status at that time (1984), and also supplied with taxonomically crucial distinguishing details of colour of dorsal cerata (3, photos by B.P. and T.K.) and scanning electron images of stylet of copulative organ (4, by A.M.). Key external distinguishing features between all four species are indicated by arrows (see Results, Discussion and supplementary information for details). Scale bars 10 μm.

This particular case is very relevant for the ongoing general ‘cryptic’ species discussion^8–10,30^ because the European nudibranch fauna is one of the best studied in the world^8,16,17,31–34^, but the three new species of *Trinchesia* presented here were never described before despite not only significant molecular divergence (Figs 1 and 2) but also multilevel morphological differences clearly defined and linked to the molecular phylogenetic data for the first time in this study (Fig. 12). For further comparison of morphological data with the molecular results see the detailed descriptions below, Figs 3–11, and the Discussion.

### Systematics

Phylum Mollusca

Order Nudibranchia Cuvier, 1817

Family Trinchesiidae Nordsieck, 1972

Genus *Trinchesia* Ihering, 1879

Type species *Doris caerulea* Montagu, 1804

## *Trinchesia caerulea* (Montagu, 1804)

(Figures 1–4, 11, 12, Tables S1–S5)

### Synonymy:

*Doris caerulea* Montagu, 1804^35^: 78, pl. 7, Figs 4, 5

*Eolidia bassi* Vérany, 1846^36^: 27

*Eolis deaurata* Dalyell, 1853^37^: 301, pl 44, Figs 8–10

*Eolis glotensis* Alder & Hancock, 1846^38^: 293

*Eolis molios* Herdman, 1881^39^: 28–29, pl 1, Figs 1–3

Non *Cuthona caerulea* sensu Schmekel & Portmann (1982)^34^, Thompson & Brown, 1984^16^ (mixture of several species).

### Material examined

#### Neotype

NE Atlantic, Mayo, Killary Harbour, Rusheen Point, Ireland (53° 37′ 31″ N 9° 51′ 26″ W), 10–20 m depth, stones with hydroids, 30.04.2017, coll. M. Larsson & B.E. Picton (ZMMU Op-646, 17 mm in length, live, preserved length 8 mm).

#### Other specimens

NE Atlantic, Wales, Pembrokeshire, Martin’s Haven, United Kingdom (51°44′15.57″ N 5°14′43.37″ W), 10–20 m depth, rocky reef, feeding on *Sertularella polyzonias*, with spawn, 02.07.2013, coll. B.E. Picton, one specimen (BELUM.Mn2018.1, 15 mm in length, live), 02.07.2013, coll. B.E. Picton. NE Atlantic, Gulen Dive Center, Norway (60° 57′27.11″ N 5° 07′ 47.10″ E), depth 15–20 m, stones, collectors T.A. Korshunova, A.V. Martynov, 05.03.2017, one specimen (ZMMU Op-622, 15 mm in length, live, ca. 9 mm in length, preserved). Mediterranean Sea, Ugljan Island, Karantun, Croatia (44° 04′ N 15° 09′ E), depth 15–20 m, collectors Đ. Iglić, A. Petani, 21.01.2018, 2 specimens (ZMMU Op-647, 21 mm live, ca. 10 mm in length, preserved). Mediterranean Sea, Ugljan Island, Karantun, Croatia (44° 04′ N 15° 09′ E), depth 15–20 m, collectors Đ. Iglić, A. Petani, 21.01.2018, one specimen (GNM Gastropoda – 9792, 15 mm live, 6 mm in length, preserved). Mediterranean Sea, Tor Paterno, Latium, Italy, one specimen (41° 40′ N 3° 12′ 20″ E), 25 m depth, collector G. Furfaro, 26.04.2013 (RM3 333). Mediterranean Sea, Tor Paterno, Latium, Italy, one specimen (41° 40′ N 3° 12′ 20″ E), 25 m depth, collector G. Furfaro, 01.05.2013 (RM3 860). Mediterranean Sea, Tor Paterno, Latium, Italy, one specimen (41° 40′ N 3° 12′ 20″ E), 25 m depth, collector G. Furfaro, 01.05.2013 (RM3 861). Mediterranean Sea, Tor Paterno, Latium, Italy, one specimen (41° 40′ N 3° 12′ 20″ E), 25 m depth, collector G. Furfaro, 26.04.2013 (RM3 309). Mediterranean Sea, Catalonia, L’Estartit, Girona, Spain (42° 02′ 32″ N 3° 13′ 38″ E), depth 16 m, stones, collector M. Pontes, 22.04.2017, one specimen (ZMMU Op-648, 20 mm live, 7 mm in length, preserved). Mediterranean Sea, Catalonia, L’Estartit, Girona, Spain (42° 02′ 32″ N 3° 13′ 38″ E), depth 16 m, stones, collector M. Pontes, 22.04.2017, one specimen (ZMMU Op-649, 9 mm live, 3 mm in length, preserved).

### Diagnosis

Body up to 21 mm with long foot corners; cerata with distinct colour zones, digestive gland basally greenish to light grayish occasionally with a narrow or diffuse dotted yellow band at the surface, an upper blue broad band, and towards the top of cerata a broad orange or yellow band; up to 12 rows of cerata, commonly four anterior rows; radular formula 57–63 × 0.1.0, penial stylet relatively short and bent at the top, seminal receptacle is 8-shaped without a long convoluted additional chamber, egg mass spiral with several whorls.

### Description

#### External morphology

The live length of the neotype is  17 mm (Fig. 3A,J). The length of adult specimens may reach 21 mm. The body is narrow. The rhinophores are smooth and 1.5–2 times longer than the oral tentacles. The cerata are relatively short, spindle-shaped. Ceratal formula of the neotype: right (2,3,4,5; anus, 5,4,3,3,2) left (1,3,4,5; anus, 5,4,4,3,2). The foot is narrow anteriorly with relatively long foot corners.

#### Colour

The basal colour is whitish to light greenish, occasionally yellow, never forming any continuous broad white line dorsally (Fig. 3). Commonly, the cerata are basally light grayish to greenish, sometimes with a narrow dotted light yellowish or orange band, then a dark to light blue broad band, and towards the top of cerata there is broad orange or yellow band. The tips of the cerata are clear with a translucent cnidosac. The tips of the rhinophores and oral tentacles sometimes have diffuse white or light yellowish or yellow pigment, but no intense orange colour. In Croatia a population of ca. 30 specimens has been observed mostly without blue pigment on the cerata, but within a few hours after their collection almost all the white pigmentation changed to blue, which varied from relatively dark to very light.

#### Anatomy

##### Digestive system

The jaws are triangularly ovoid (Fig. 4C). The masticatory processes of the jaws bear a single row of conspicuous low denticles (Fig. 4D). The radular formula in two studied specimens is 57–63 × 0.1.0. The radular teeth are yellowish. The central tooth is broad, with low cusp and 6–8 lateral denticles, including smaller intercalated denticles that may occur in different parts of the tooth (Fig. 4A,B).

##### Reproductive system

(Fig. 11A). The ampulla is massive and swollen (Fig. 11A, am). The prostate is a convoluted tube (Fig. 11A, pr). The prostate transits to a penial sheath, which contains a conical penis with a relatively short, chitinous stylet, strongly curved at the top (Figs 4E–H, 11A, ps). A supplementary (“penial”) gland inserts into the base of the penis and is attached to the penial sheath along of its most length (Fig. 11A, pg). The seminal receptacle is an 8-shaped structure, comprising two reservoirs partly inserted into each other (Fig. 11A, rs). The female gland mass includes mucous and capsular glands (Fig. 11A, fgm).

### Description of egg masses

The egg mass is a white or very pale pinkish spiral cord forming about 3 whorls or an irregular cord. It may contain 1000 eggs or more.

### Distribution and habitats

Reliably confirmed from Britain and Ireland, Norway, and the Mediterranean Sea (Croatia, Italy and Spain). Found in stony, relatively shallow areas, often covered with various algae, usually at 10–25 m depth, occasionally at only 1–2 m. Feeds on hydrozoans of the genus *Sertularella* (*S*. *polyzonias* (L., 1758)) but also on *S*. *crassicaulis* (Heller, 1868) and *S*. *gayi* (Lamouroux, 1821) for which it has a strong preference^40^, and it is also reported feeding on *Eudendrium racemosum* (Cavolini, 1785)^41^, *Hydrallmania falcata* (L., 1758) and *Halecium halecinum* (L., 1758)^16^. We provide references to the potential food sources here, but this information should be used carefully since food associated with *T*. *caerulea* may now be the food preference of one of the other *T*. *caerulea* complex species. In the French Mediterranean, *T*. *caerulea* was recorded as feeding on *S*. *crassicaulis*. In the present study *T*. *caerulea* was confidently found commonly in L’Estartit (Catalonia) area as associated with *S*. *polyzonias* and much less frequently on *Eudendrium racemosum*.

### Remarks

#### Morphological differences

*T*. *caerulea* can be distinguished from the partly sympatric (in the NE Atlantic) *T*. *cuanensis* sp. n. by this set of morphological data: 1). Greenish to light grayish basis of the cerata, not blackish to dark grayish as in *T*. *cuanensis* sp. n. 2). Absence on the cerata of a narrow blackish band above the broad blue band, which is present in *T*. *cuanensis* sp. n. 3). Long anterior foot corners, not short ones as in *T*. *cuanensis* sp. n.; 4). Relatively short penial stylet, whereas *T*. *cuanensis* sp. n. has an extraordinarily long stylet. *T*. *caerulea* can be distinguished from the partly sympatric, predominantly Mediterranean species, *T*. *morrowae* sp. n., by this set of morphological data: 1). Greenish to light grayish basis of cerata, not light grayish to yellowish as in *T*. *morrowae* sp. n.; 2). Absence of a distinct white dorsal line and thin white lateral lines, which are instead always present in *T*. *morrowae* sp. n.; 3). White opaque or light yellowish tips of rhinophores and oral tentacles, but not orange ones as in *T*. *morrowae* sp. n. 4). Long anterior foot corners, not just angular projections as in *T*. *morrowae* sp. n.; 5). The penial stylet is curved towards the tip, whereas in *T*. *morrowae* sp. n. it is curved rather basally; 6). *T*. *caerulea* has up to two times the adult body size of *T*. *morrowae* sp. n. *T*. *caerulea* can be distinguished from the exclusively Black Sea species *T*. *diljuvia* sp. n. by this set of morphological data: 1). Presence of distinct colour zones on the cerata, which are absent in *T*. *diljuvia* sp. n.; 2). *T*. *caerulea* has up to five times larger adult body size compared to *T*. *diljuvia* sp. n.; 3). Absence of a white dorsal line, which is always present in *T*. *diljuvia* sp. n.; 3). Long anterior foot corners, which are completely absent in *T*. *diljuvia* sp. n.; 5). Precise shape of the penial stylet is different between *T*. *caerulea* and *T*. *diljuvia* sp. n.

#### Molecular differences

Minimum uncorrected COI p-distances between the T. *caerulea* neotype specimen and *T*. *cuanensis* sp. n., *T*. *morrowae* sp. n. and *T*. *diljuvia* sp. n. specimens are 7.36%, 11.11%, and 12.05% respectively. See also Discussion, Fig. 12 for integration of molecular phylogenetic and morphological data, and supplementary information, Tables S1–S5.

## *Trinchesia cuanensis* sp. n.

(Figures 1, 2, 5, 6 and 11, 12, Tables S1–S5).

### Synonymy

*Cuthona caerulea* sensu lato auct., e.g. Thompson & Brown (1984)^16^, Schmeckel & Portmann (1982)^34^ (mixed up with *Doris caerulea* Montagu, 1804).

### Material examined

#### Holotype

NE Atlantic, Strangford Lough, Down, Ballyhenry, Northern Ireland, United Kingdom (54°23′22.2″ N 5°34′23.4″ W), 15–20 m depth, coll. B.E. Picton, 24.05.2014 (ZMMU Op-650, 15 mm in length, live, 7 mm in length, preserved). Paratypes. NE Atlantic, Strangford Lough, Portaferry, Northern Ireland, United Kingdom (54° 22,0′ N 05°32,0′ W), ca. 10 m depth, coll. B.E. Picton, 22.05.2014, one specimen (GNM Gastropoda – 9054, ca. 9 mm in length, preserved). NE Atlantic, Strangford Lough, Down, Ballyhenry, Northern Ireland, United Kingdom (54°23′22.2″ N 5°34′ 23.4″ W), 10–25 m depth, coll. B.E. Picton, 24.05.2014, one specimen (ZMMU Op-655, 15 mm in length, live, 8 mm in length, preserved). NE Atlantic, Portrush, Antrim, S of Little Skerry, Northern Ireland, United Kingdom (55°′12′59.238″ N 6° 38′39.292″ W), 24.3 m max depth, coll. B.E. Picton, 23.08.2006, three specimens (BELUM.Mn.2018.2, 13 mm in length, live, 5 mm in length, preserved). Bohuslän, outside of Smögen, Sweden (58° 21,00′ N 11°12,00′ E), 10–20 m depth, coll. Klas Malmberg, 02.05.2015, one specimen (GNM Gastropoda – 9243, 2 mm in length, preserved).

### Zoobank registration

urn:lsid:zoobank.org:act: 77629514-DDB5-4757-86FE-78DDD7672563.

### Etymology

After Lough Cuan, an alternative name for Strangford Lough in Northern Ireland, where many marine biological studies have been undertaken over the past 100 years.

### Diagnosis

Body up to 15 mm (live), cerata with distinct colour zones, digestive gland basally dark grayish to blackish with commonly a narrow or reduced dotted orange or yellowish band, then narrower black zone, then a blue broad band, and towards the top of cerata there is broad orange or yellow band, up to 10 rows of cerata, commonly four anterior ceratal rows, radular formula 64 × 0.1.0, penial stylet extraordinarily long, rounded seminal receptacle with a long convoluted additional chamber, egg mass spiral with several whorls.

### Description

#### External morphology

The length of the preserved holotype is 7 mm (Fig. 5). The live length up to 15 mm. The body is narrow. The rhinophores are smooth and 1.5–2.5 times longer than the oral tentacles. The cerata are relatively short, spindle-shaped. Ceratal formula of the holotype: right (3,4,5,6; anus, 7,5,4,3,3) left (2,4,5,6; anus, 8,5,5,3,3). The foot is narrow, anteriorly with short foot corners.

#### Colour

The basal colour is whitish to light greenish, sometimes with some diffuse pigment of a similar colour scattered over the body, but never forming any continuous broad white line dorsally (Fig. 5). The cerata are basally blackish to dark grayish (no green colour) with a narrow dotted orange or a yellowish band, then a dark blue broad band, followed by a narrow dark band, and towards the top of cerata there is broad orange or yellow band. The tips of the cerata are clear with a translucent cnidosac. The tips of the rhinophores and oral tentacles have diffuse white pigment, which is sometimes yellowish, but never of an intense orange colour.

#### Anatomy

##### Digestive system

The jaws are triangularly ovoid (Fig. 6B). The masticatory processes of the jaws bear a single row of inconspicuous low denticles (Fig. 6C). The radular formula is 64 × 0.1.0. The radular teeth are yellowish. The central tooth is broad, with low cusp and 7–8 lateral denticles, including smaller intercalated denticles near the cusp (Fig. 6A).

##### Reproductive system

(Fig. 11B). The ampulla is massive and swollen (Fig. 11B, am). The prostate is a highly convoluted tube (Fig. 11B, pr). The prostate transits to a penial sheath, which contains a conical penis with an extremely long, chitinous stylet, slightly curved at the top (Figs 6D–H, 11B, ps). A supplementary (“penial”) gland inserts into the base of the penis and attaches to the penial sheath along most of its length (Fig. 11B, pg). The seminal receptacle is a complex structure with long narrow stalk, large rounded reservoir and long convoluted narrow special supplementary reservoir inserted into large rounded one (Fig. 11B, rs). The female gland mass includes mucous and capsular glands (Fig. 11B, fgm).

### Description of egg masses

The egg mass is a spiral cord forming a minimum of 2 whorls, or an irregular cord. Number of eggs is commonly more than 300.

### Distribution and habitats

Confirmed records in Ireland, United Kingdom and Swedish west coast. Found in stony, relatively shallow areas, at 10–20 m depth. Feeds on hydrozoans *Sertularella polyzonias*, and possibly other *Sertularella* species.

### Remarks

#### Morphological differences

*T*. *cuanensis* sp. n. can be distinguished from the partly sympatric (both are common in the North Atlantic) type species of the genus, *T*. *caerulea*, by this set of morphological data: 1). Blackish to dark grayish basis of the cerata, not greenish to light grayish as in *T*. *caerulea*; 2). Presence on the cerata of a narrow blackish band above the broad blue band, but not in *T*. *caerulea*; 3). Short anterior foot corners, not long as in *T*. *caerulea*; 4). Extraordinarily long penial stylet (not normal for the family Trinchesiidae), whereas *T*. *caerulea* has a normal, short one. *T*. *cuanensis* sp. n. can be distinguished from the partly sympatric, predominantly Mediterranean species, *T*. *morrowae* sp. n., by this set of morphological data: 1). Blackish to dark grayish basis of the cerata, not light grayish to yellowish as in *T*. *morrowae* sp. n.; 2). Presence on the cerata of a narrow blackish band above the broad blue band, not present in *T*. *morrowae* sp. n.; 3). Absence of a white dorsal line and thin white lateral lines, which are instead always present in *T*. *morrowae* sp. n.; 4). Short anterior foot corners, not just angular projections as in *T*. *morrowae* sp. n.; 5). Extraordinarily long penial stylet, whereas *T*. *morrowae* sp. n. has a normal short one. *T*. *cuanensis* sp. n. can be distinguished from the exclusively Black Sea species *T*. *diljuvia* sp. n. by this set of morphological data: 1). Presence of distinct colour zones on the cerata, which are absent in *T*. *diljuvia* sp. n.; 2). Absence of a white dorsal line, which is always present in *T*. *diljuvia* sp. n.; 3). Short anterior foot corners, which are completely absent in *T*. *diljuvia* sp. n.; 4). Extraordinarily long penial stylet, whereas *T*. *diljuvia* sp. n. has a relatively short one.

#### Molecular differences

Minimum uncorrected COI p-distances between *T*. *cuanensis* sp. n. type specimen and *T*. *caerulea*, *T*. *morrowae* sp. n. and *T*. *diljuvia* sp. n. specimens are 7.98%, 12.05%, and 13.77% respectively. See also Discussion, Fig. 12 for integration of molecular phylogenetic and morphological data, and supplementary information, Tables S1–S5.

## *Trinchesia morrowae* sp. n.

(Figures 1, 2, 7, 8, 11, 12, Tables S1–S5).

### Synonymy

*Cuthona caerulea* auct., e.g. Thompson & Brown (1984)^16^, Schmeckel & Portmann (1982)^34^, Picton & Morrow (1994)^31^ non *Doris caerulea* Montagu, 1804.

### Material examined

#### Holotype

Mediterranean Sea, Catalonia, L’Estartit, Girona, Spain (42° 02′ 32″ N 3° 13′ 38″ E), depth 16 m, stones, collector M. Pontes, 22.04.2017 (ZMMU Op-651, 9 mm live, 3.2 mm in length, preserved). Paratypes. Mediterranean Sea, Banyuls-sur-Mer, France (42° 28′ 53.0″ N 3° 08’ 17″ E), depth 5–12 m, stones, collector T.A. Korshunova, A.V. Martynov, 08.09.2010, two specimens (ZMMU Op-653, ca. 8 mm live, ca. 3 mm in length, preserved). Mediterranean Sea, Banyuls-sur-Mer, France (42° 28′ 53.0″ N 3° 08′ 17″ E), depth 5–12 m, rocks, stones, collector T.A. Korshunova, A.V. Martynov, 10.09.2010, one specimen (ZMMU Op-535, 5 mm live, 3.5 mm in length, preserved). Mediterranean Sea, Banyuls-sur-Mer, France (42° 28′ 53.0″ N 3° 08′ 17″ E), depth 5–12 m, rocks, stones, collector T.A. Korshunova, A.V. Martynov, 09.09.2010, one specimen (ZMMU Op-656, 7 mm live, 2.5 mm in length, preserved). Mediterranean Sea, Catalonia, L’Estartit, Girona, Spain (42° 02′ 32″ N 3° 13′ 38″ E), depth 16 m, stones, collector M. Pontes, 22.04.2017, one specimen (ZMMU Op-652, 9 mm live, 3.2 mm in length, preserved).

### Zoobank registration

urn:lsid:zoobank.org:act: D7DB7FFB-F6B1-4A67-8DF4-A1D0F05963A7.

### Etymology

After Christine Morrow, co-author of “A Field Guide to the Nudibranchs of the British Isles” (1994), who sequenced a specimen of *Trinchesia cuanensis* which first revealed the presence of two species of this complex in the NE Atlantic.

### Diagnosis

Body up to ca. 10 mm, white dorsal line, cerata with distinct colour zones, digestive gland basally light grayish to yellowish, then a usually broad distinct dotted orange band, then narrower black zone, then a blue broad band, then, compared to *T*. *cuanensis* sp. n., there is no narrow black band and towards the top of cerata there is broad orange band, three, rarely four anterior ceratal rows, radular formula 55–57 × 0.1.0, penial stylet short and considerably curved, seminal receptacle is oval without additional chamber, egg mass a short spiral.

### Description

#### External morphology

The length of the holotype is 9 mm (live, Fig. 7). The length of adults may be up to 11 mm. The body is narrow. The rhinophores are smooth and ca. 1.5 times longer than the oral tentacles. The cerata are relatively short, spindle-shaped. The ceratal formula of the holotype: right (2,3,3; anus, 3,3,2,2,1) left (2,3,4; 4,3,2,2,1). The foot is narrow anteriorly without real foot corners, only angular processes.

#### Colour

The basal colour is whitish and a distinct thick white line runs dorsally, thinner white lines run laterally (Fig. 7). The cerata are basally light grayish to yellowish with a broad dotted orange band, then a narrower black zone, then a blue broad band, and towards the top of cerata there is broad orange band (there is no distinct black line in between the blue and orange upper zones). The tips of the cerata are clear with a translucent cnidosac. The tips of the rhinophores and oral tentacles have an intense orange pigment.

#### Anatomy

##### Digestive system

The jaws are triangularly ovoid (Fig. 8C) The masticatory processes of the jaws bear a single row of low, inconspicuous denticles (Fig. 8D). The radular formula in the holotype is 55–57 × 0.1.0. The radular teeth are yellowish. The central tooth is broad, with a low cusp and 4–7 lateral denticles, including smaller intercalated denticles (Fig. 8A,B).

##### Reproductive system

(Fig. 11C). The ampulla is massive and swollen (Fig. 11C, am). The prostate is a highly convoluted tube (Fig. 11C, pr). The prostate transits to a penial sheath, which contains a conical penis with a short chitinous stylet, slightly curved at the top (Figs 8E,F, 11C, ps). A supplementary (“penial”) gland inserts into the base of the penis and attaches to the penial sheath along most of its length (Fig. 11C, pg). The seminal receptacle is a relatively simple structure with a widened stalk and a small bent reservoir (Fig. 11C, rs). The female gland mass includes mucous and capsular glands (Fig. 11C, fgm).

### Description of egg masses

The egg mass is a cream coloured spiral cord of at least 2–2.5 whorls, or a regular cord. Number of eggs less than 100.

### Distribution and habitats

Predominantly known from various locations in the Mediterranean Sea, from Greece to the Western basin, but may also reach the southern part of the UK. Occurs in all the Iberian Peninsula coastal areas, including both Portugal and Spain. Found in stony, relatively shallow areas, from 1 to 25 m depth, also in *Posidonia oceanica* (L.) meadows. Feeds on *Sertularella* spp. hydroids, possibly on *Sertularia perpusilla* Stechow, 1919, and also *Stylactis inermis* Allman, 1872.

### Remarks

#### Morphological differences

*T*. *morrowae* sp. n. can be distinguished from the partly sympatric (both are common in the North Atlantic and in the Mediterranean Sea) type species of the genus, *T*. *caerulea*, by this set of morphological data: 1). Light grayish- to yellow basis of the cerata, not greenish to light grayish as in *T*. *caerulea*; 2). Presence of a distinct lower orange band on the cerata, not a commonly reduced and diffuse one as in *T*. *caerulea*; 3). Presence of a thick white dorsal line and thin white lateral lines, which are always absent in *T*. *caerulea*; 4). Presence of commonly three, rarely four, anterior ceratal rows in *T*. *morrowae* sp. n., whereas there are commonly four in *T*. *caerulea*; 5). Angular anterior projections of the foot, not long foot corners as in *T*. *caerulea*; 6). Shape of the penial stylet, which is bent at the top in *T*. *caerulea*, but bent at the basis or middle in *T*. *morrowae* sp. n. 7). *T*. *morrowae* sp. n. has an adult body size up to half as large as *T*. *caerulea*.*T*. *morrowae* sp. n. can be distinguished from the partly sympatric (present only in the North Atlantic) *T*. *cuanensis* sp. n. by this set of morphological data: 1). Light grayish to yellowish basis of the cerata, not blackish to dark grayish as in *T*. *cuanensis* sp. n.; 2). Presence of a distinct lower orange band on the cerata, not a commonly diffuse reduced one as in *T*. *cuanensis* sp. n. 3). Presence of a thick white dorsal line and thin white lateral lines, which is always absent in *T*. *cuanensis* sp. n.; 4). Presence of commonly three, rarely four, anterior ceratal rows in *T*. *morrowae* sp. n., whereas there are commonly four in *T*. *cuanensis* sp. n.; 5). Angular anterior projections of the foot, not short foot corners as in *T*. *cuanensis* sp. n.; 6). Length of the penial stylet, which is short in *T*. *morrowae* sp. nov., but is extremely long in *T*. *cuanensis*. 7). *T*. *morrowae* sp. n. has an adult body size up to 2/3 that of *T*. *cuanensis* sp. n. *T*. *morrowae* sp. n. can be distinguished from the exclusively Black Sea species *T*. *diljuvia* sp. n. by this set of morphological data: 1). Presence of distinct colour zones on the cerata, which are absent in *T*. *diljuvia* sp. n.; 2). Presence of commonly three, rarely four, anterior ceratal rows in*T*. *morrowae* sp. n., whereas there are commonly two in *T*. *diljuvia* sp. n.; 3). Short anterior foot corners, which are completely absent in *T*. *diljuvia* sp. n.; 4). Shape and relative length of the penial stylet, which is short in*T*. *morrowae* sp. n. and relatively longer in *T*. *diljuvia* sp. n.

#### Molecular differences

Minimum uncorrected COI p-distances between the *T*. *morrowae* sp. n. type specimen and *T*. *caerulea*., and *T*. *cuanensis* sp. n specimens are 11.27%, and 11.42% respectively. COI p-distances between *T*. *morrowae* sp. n. and *T*. *diljuvia* sp. n. ranged from 2.98–4.23%, whereas intraspecific divergence ranged from 0.16 - 2.18% in *T*. *morrowae* sp. n. and 0.16% in *T*. *diljuvia* sp. n. which is smaller than the interspecific differences between these species. Intraspecific divergence in *T*. *morrowae* sp. n. may indicate that *T*. *morrowae* sp. n. is still undergoing the process of evolutionary divergence. This supports the scenario of an extremely rapid evolution of the Mediterranean species *T*. *morrowae* sp. n. (or its closely related ancestral species) in *T*. *diljuvia* sp. n. See also Discussion, Fig. 12 for integration of molecular phylogenetic and morphological data, and supplementary information, Tables S1–S5.

## *Trinchesia diljuvia* sp. n.

(Figures 1, 2, 9–12, Tables S1–S5).

### Material examined

#### Holotype

Black Sea, Yalta region, Russia (44° 29′ N 34° 10′ E), depth 1–2 m, stones, collector T.A. Korshunova, A.V. Martynov, 24–26.07.2004, (ZMMU Op-642, 4.5 mm live, ca. 2 mm in length, preserved). Paratypes. Black Sea, Yalta region, Russia (44° 29′ N 34° 10′ E), depth 1–2 m, stones, collector T.A. Korshunova, A.V. Martynov, 28.07.2004, 1 specimen, dissected (ZMMU Op-643, ca. 3 mm live, ca. 1.5 mm in length, preserved). Black Sea, Yalta region, Russia (44° 29′ N 34° 10′ E), depth 1–2 m, stones, collector T.A. Korshunova, A.V. Martynov, 31.07.2004, 1 specimen, dissected (ZMMU Op-645, ca. 3.5 mm live, ca. 2 mm in length, preserved).

### Zoobank registration

urn:lsid:zoobank.org:act: C9228E8B-DF2C-46A0-9E78-038ED6B87D7B.

### Diagnosis

Body up to 4.5 mm, white dorsal line, no colour zones on light brownish (with traces of bluish pigment) cerata, commonly two, maximum three, anterior ceratal rows, radular formula 27–34 × 0.1.0, penial stylet short slightly curved, seminal receptacle is rounded without any additional chamber, egg mass short semi-spiral.

### Etymology

Combination of Latin, *diluviim*, deluge, inundation and *juvenilis*, youthful, alluding to the paedomorphic appearance of the new species and speciation during the formation of the modern Black Sea after the Holocene meltwater events (see Discussion).

### Description

#### External morphology

The length of the holotype is 4.5 mm (Fig. 9). The length of adults reaches 4.5 mm. The body is narrow. The rhinophores are smooth and 1.5 times longer than the oral tentacles. The cerata are short, distinctly spindle-shaped. Ceratal formula of the holotype: right (1,2,3; anus, 3,2,2,1) left (1,2,3; 3,2,2,1). The foot is narrow anteriorly without foot corners or angular processes.

#### Colour

The basal colour is whitish; a distinct thick white line runs dorsally with median expansion (Fig. 9). The cerata and digestive gland lack distinct orange and bright blue colour zones as seen in the previous three species. Most parts of the cerata are dark grayish to light brownish or light greenish and covered with white or sometimes with light bluish dispersed pigment. Sometimes towards the top of the cerata there is a thin darker band between the brownish zone and white covering. The tips of the cerata are clear with a pinkish cnidosac. The tips of rhinophores and oral tentacles are encrusted with dense white-yellowish to greenish pigment.

#### Anatomy

##### Digestive system

The jaws are triangularly ovoid (Fig. 10C) The masticatory processes of the jaws bear a single row of low, inconspicuous denticles (Fig. 10D). The radular formula in the holotype is 27–34 × 0.1.0. The radular teeth are yellowish. The central tooth is narrow, elongated, with low cusp and 4–7 lateral denticles, including smaller intercalated denticles) (Fig. 10A,B).

##### Reproductive system

(Fig. 11D). The ampulla is moderately short and swollen (Fig. 11D, am). The prostate is a slightly convoluted tube (Fig. 11D, pr). The prostate transits to a penial sheath, which contains a conical penis with a basally slightly curved chitinous stylet (Figs 10E, 11D, ps). A supplementary (“penial”) gland inserts into base of the penis (Fig. 11D, pg). The seminal receptacle is small, rounded, and on a stalk (Fig. 11D, rs). The female gland mass includes mucous and capsular glands (Fig. 11D, fgm).

### Description of egg masses

The egg mass is a small semi-spiral cord with only a few eggs (ca. 20) (Fig. 9G).

### Distribution and habitats

Black Sea, Yalta region only, not found in the Mediterranean or other regions. Found in stony, shallow areas, around 1–2 m depth, on *Sertularella* spp. hydroids.

### Remarks

#### Morphological differences

*T*. *diljuvia* sp. n. can be distinguished from the North Atlantic and Mediterranean species *T*. *caerulea* by this set of morphological data: 1). Absence of distinct colour zones on the cerata, which are present in *T*. *caerulea*; 2). Presence of a white dorsal line in *T*. *diljuvia* sp. n., which is absent in *T*. *caerulea*; 3). Presence of commonly two, rarely three, anterior ceratal rows in*T*. *diljuvia* sp. n., whereas there are commonly four in *T*. *caerulea*; 4). Absence of anterior foot corners in*T*. *diljuvia* sp. n., which are present and long in *T*. *caerulea*; 5). Shape and relative length of the penial stylet, which is relatively longer in *T*. *diljuvia* sp. n. and relatively shorter in *T*. *caerulea*. *T*. *diljuvia* sp. n. can be distinguished from the exclusively North Atlantic species *T*. *cuanensis* by this set of morphological data: 1). Absence of distinct colour zones on the cerata, which are present in *T*. *cuanensis* sp. n.; 2). Presence of a white dorsal line in *T*. *diljuvia* sp. n. which is absent in *T*. *cuanensis* sp. n.; 3). Presence of commonly two, rarely three, anterior ceratal rows in *T*. *diljuvia* sp. n., whereas there are commonly four in *T*. *cuanensis* sp. n.; 4). Absence of anterior foot corners in *T*. *diljuvia* sp. n., which are present and short in *T*. *cuanensis* sp. n.; 5). Shape and relative length of the penial stylet, which is relatively very short in *T*. *diljuvia* sp. n. compared to the extremely long stylet in *T*. *cuanesis* sp. n. *T*. *diljuvia* sp. n. can be distinguished from the predominantly Mediterranean species *T*. *morrowae* sp. n. by this set of morphological data: 1). Absence of distinct colour zones on the cerata, which are present in *T*. *morrowae* sp. n.; 2). Presence of commonly two, rarely three, anterior ceratal rows in *T*. *diljuvia* sp. n., whereas there are commonly three in *T*. *morrowae* sp. n.; 3). Absence of anterior foot corners in *T*. *diljuvia* sp. n., whereas distinct angular projections are present in*T*. *morrowae* sp. n..; 4). Shape and relative length of the penial stylet, which is relatively longer in *T*. *diljuvia* sp. n. and relatively shorter in *T*. *morrowae* sp. n.

#### Molecular differences

Minimum uncorrected COI p-distances between the *T*. *diljuvia* sp. n. type specimen and *T*. *caerulea*, *T*. *cuanensis* sp. n. and *T*. *morrowae* sp. n., specimens are 11.89%, 12.99%, and 2.98% respectively. See also Discussion, Fig. 12 for integration of molecular phylogenetic and morphological data, and supplementary information, Tables S1–S5.

## Discussion

### The particular case of a nudibranch species complex and the general ‘cryptic species’ problem

This case of a colourful nudibranch species that represents an apparently very cryptic *T*. *caerulea* complex from Britain and Ireland through continental Europe and the Mediterranean to the Black Sea, i.e., with a putative low degree of morphological disparity in the sense of Struck *et al*.^9^, in turn reveals both stable external and internal features (Figs 1–12, Table S5, where external and internal distinguishing features for all four species are summarized). Prior to this study (*T*. *caerulea* was described initially from the UK more than 200 years ago), these putative morphs within the single *T*. *caerulea* species were always claimed to be “internally the same/similar”^16,34^, thus fulfilling the current definition^9^ of a ‘cryptic’ species.

To challenge this notion that ‘cryptic’ species can only be defined *a posteriori* by the molecular data^18,19^ we specifically included the illustration from Thompson & Brown (1984)^16^ (Fig. 12) to show that the separate species described here are recognizable even from the external morphological features (Figs 1, 12), but were considered at that time to be just a variation of *T*. *caerulea*, i.e., in this case this species complex could be regarded as ‘pseudocryptic’ prior to a molecular study. In this respect, it could be argued that we are confident about the separate species status of new species because we now have molecular data, whereas previous authors did not. However, the two common sympatric British and Irish species involved in this study, though they have a putatively similar/identical colour pattern, show very different characters at another level [i.e., penial stylets (Fig. 12)] which were overlooked in all previous studies but this trait implies a great obstacle during potential inter-species copulation. If these substantial differences in penial stylets would have been detected during previous morphological studies^16^, the question about their separate species status would have been raised much earlier. These data immediately contradict the updated definition of ‘cryptic species’^9^ which requires ‘cryptic species’ to be genetically isolated but morphologically non-distinguishable. Despite that this species complex was potentially morphologically distinguishable using fine details even in the pre-molecular era^16^ (and hence can be classified a ‘pseudocryptic’ species^18^, or ‘normal’ species not *a posteriori* to the molecular data but *a priori*), it remained almost absolutely ‘cryptic’ until recently because smaller distinguishing taxonomic units were not defined at that time and researchers decided they could not plausibly grant them a separate species status under what, at the time, was a dominant morphological “lumping” taxonomic framework. Therefore, this species complex can be considered ‘cryptic’, ‘pseudocryptic’ and ‘non-cryptic’ at the same time. The novel data on multilevel diversity from a particular species complex presented here, therefore, confirm that the general ‘cryptic species’ concept is far from being a satisfying solution and that the commonly used ‘cryptic species’ concept needs to be reconsidered.

### The term ‘cryptic species’ has a vague definition and includes non-cryptic diversity

Struck *et al*.^9^ attempted to provide a stricter definition of cryptic species and proposed two main components of an updated definition: “diverged genotypic clusters of individuals (reflecting reproductive isolation) that do not form diagnostic morphological clusters” and the temporal dimension of “lower degrees of phenotypic (or more specifically morphological) disparity than non-cryptic relatives”. The central proposal of the new updated ‘cryptic’ species definition^9^ has a deficiency in a heavy dependence on a detailed study of a species/organism and an existing taxonomic framework of a particular group. For example, most of the European nudibranch species of the genus *Dendronotus* were just a single, very complicated and non-diagnosable ‘cryptic’ species, even for experts, but a recent study revealed that it is possible to provide not only molecular values, but also morphological differences for every species in the complex^8^. Furthermore, the requirements^9^ for a cryptic species to be morphologically non-distinguishable but genetically isolated is also controversial, since there are many examples where genetically separate species either are similar morphologically (as is the case for the blue mussel species complex) or considerably different as is the case for dolphins and whales, but in both groups numerous cases of genetic introgression and/or presence of fertile hybrids have been demonstrated^42,43^. As an answer to a recent proposal to consider ‘cryptic’ species rather as a temporary term^8^ some authors continued to argue for validity of the cryptic species^9^ concept and mentioned previous studies of such different animal groups as lizards, annelids, molluscs, cnidarians, and mammals as examples of putatively “uniform” morphology among different species, which, however, under scrutiny may demonstrate diagnosable morphological taxa. Notably, in the example with an annelid species^44^ it was indicated that “the cryptic species complex of *Stygocapitella subterranea* is not as cryptic as assumed as the Australian populations are morphologically and genetically different”.

If we claim that we cannot find any morphologically diagnosable structures in a ‘cryptic’ species complex, this would imply that we have exhaustively studied organism features and are able to unambiguously present all existing ‘macromorphological’ and ‘micromorphological’ features for a given organism. This is obviously impossible at the current level of our technology and knowledge. The putative strict distinction between morphological and molecular levels of organisms, on which the cryptic species concept is largely relying, loses clarity when we proceed to the tissue or cellular ‘micromorphological’ level. To the utmost degree, morphologically absolutely identical “body doubles”^3^ can be formed only on the basis of zygote clones that automatically imply its identical genetic structure. If we instead discovered putative “species clones” but with some considerable genetic differences, this most likely suggests that we have missed some morphological differences during a study. Therefore, it is biologically impossible for species to be “physically indistinguishable from each other”^1,18,19^ since even clones can be different from each other because of epigenetic mechanisms^45–48^.

In practice there is an array of various cases, from very highly similar external species, for which authors reported that they have not yet found confident morphological differences, e.g., in American lizards^49^, Japanese microsnails^50^, coryphellid nudibranchs of the genus *Gulenia*^25^ to species which are externally difficult to distinguish (but still possible) but internally demonstrate stable distinct characters, e.g., in the European nudibranch species of the genus *Dendronotus*^8^. This series continues with species which demonstrate some overall similarity and appear cryptic but under more detailed study turn out to demonstrate reliable and stable external and internal differences, like a polychaete species complex^44^ and the present case of the four *Trinchesia* species complex. Finally, there are the cases when the term ‘cryptic’ species was applied to externally diagnosable species (e.g. in the echinoderm ophiuroids^13^). Thus, a considerable amount of non-cryptic diversity is still currently included under the ‘cryptic species’ concept. It is therefore likely that if we really would like to make a distinction between ‘species’ and ‘cryptic’ species we will need a much more differentiated system of terminology than this simplistic pair ‘cryptic’ vs. ‘non-cryptic’. If we use the term ‘cryptic species’ conditionally and for the purpose of this discussion only, for example, additional terms might be ‘true cryptic species’ (morphological differences have not yet been found), ‘semi-cryptic species’ (morphological differences are very difficult to present), ‘quasi-cryptic species’ (morphological differences are relatively easy to present) and finally a ‘false cryptic species’ (morphological differences are obvious, but for some reason were missed or not highlighted in previous studies). However, while we can use such terminology for a preliminary sorting of multilevel diversity, quite obviously, the number of such various intermediate terms would grow exponentially, and it may be difficult to apply to other taxa since many cases may represent their own unique combination of genetic divergence/uniformity and morphological external and internal similarity/disparity. Remarkably, the last putative term in this series, ‘false cryptic species’ actually does not differ fundamentally from the basic term ‘species’, and the term ‘pseudocryptic species’ was previously applied to cases where it is possible to show at least some morphological differences^4,18^. It has already been noted that the line between cryptic and pseudocryptic “is not sharp”^51^, but these terms have been loosely applied until the meaning is lost^52^. Notably, the term ‘cryptic species’ has been applied^53^ to evaluate a nudibranch species revealed in a previous study^54^ to be “very similar to *Limacia clavigera*” yet with “consistent morphological and external differences between the two species examined” but without any corresponding molecular data for the morphologically-only defined “cryptic” *Limacia iberica* Caballer *et al*., 2016^54^ species. Yet a previous proposal^19^ noted that cryptic species are, by definition, molecularly different species that can only turn out to be morphologically distinguishable ‘pseudocryptic’ species *a posteriori*. Thus, such highly inconsistent usage of the term ‘cryptic’ species confirms the most recent notion of Heethoff^10^ that the ‘cryptic’ species concept currently represents both conceptual and terminological chaos.

Taking also into consideration that one of the united definitions of the term ‘species’ defines it as only being separately evolved metapopulations, which are not required to be morphologically different or even reproductively isolated^55^, this in turn implies that any species at its origin is already completely ‘cryptic’. Indeed, many so called ‘cryptic’ species represent recently diverged lines and therefore their overall similarity may rely on significant similarity in the developmental genes responsible for the formation of the morphological structures, rather than on some mutations that may occur in the housekeeping genes which are commonly used for taxonomic purposes. More wide applications of full genomic/transcriptomic comparison may therefore potentially contribute to finding not only morphological but also high genetic similarity within recently diverged closely related lineages. Hence the term ‘super-cryptic’ species would be needed for the species without morphological, genetic or ecological differences which though probable, is only theoretically possible.

Therefore, these apparent ‘cryptic’ species with “low morphological disparity” instead turned out to be a species which previously was “insufficiently studied” at different levels of morphological organisation. Thus, at a given time technological and scientific achievements allow us to understand some species and present their descriptions in more and more detail using the dominant paradigm at the time for species descriptions that may easily lead to some well-distinguishable features being missed. As a result, when a new study uncovers new details, a species can be easily considered as not ‘cryptic’ or ‘pseudocryptic’ anymore, but just a normally diagnosable species. This is the exact situation of the present discovery of well-diagnosable morphological patterns at different levels in the genus *Trinchesia*, so that what was a totally cryptic and non-diagnosable complex a few years ago is now four well-diagnosable and non-cryptic separate species. Even if complexes in other organism groups that are apparently even more ‘cryptic’ than the present case^25,49,50,56^ still exist, and potentially can be discovered further within that nudibranch species complex, this does not mean that after some time we will not be able to study their morphological structures using, for example, more advanced micro CT or a similar technique available to future generations, including potential detailed scanning of living animals, and also discovering fine details of their ecology, etc., and will be able therefore to elaborate and define well-diagnosable taxonomic units, as we have presented here for the British and Mediterranean *Trinchesia* species. Another nudibranch species that previously was incorrectly identified as “*T*. *caerulea*” from the Caribbean Sea was shown to be considerably different from the true *T*. *caerulea* both by morphological and molecular data^57^. Morphologically distinguishing characters, therefore, should not be identified only *a posteriori*^18^ but should have at least similar weight with the molecular ones. A molecular study may promote discovery of a hidden diversity within a group, but a robust taxonomic framework can be built only in an agreement with morphological data, even if such a task is difficult and requires a more detailed study such as those undertaken for the other nudibranchs^8,58^, when a considerable amount of external interspecific variability is present and some species were externally nearly identical to other related but different species. In turn, in a completely opposite situation, the presence of morphologically well-recognized species which have a very low degree of molecular divergence from related species^1,59,60^ clearly indicates the necessity of restoring the significance of morphology to the diagnoses of species in the form of detailed morphological data.

### Fine-scale multilevel framework for studying species

When we discovered several new species within a complex traditionally assigned to a single species, we created a new morphological and molecular framework that defined much smaller taxonomically distinguishable units than were previously used. Because life is hierarchically organized^61^ there is a multilevel diversity in character evolution^8^, and correspondingly, further smaller taxonomically relevant morphological and molecular concordant units can be discovered within previously selected larger taxonomic groups. This process can be potentially quite endless, or at least take a considerable amount of time, while we set an increasingly narrower scale for such morphological and molecular taxonomic units (those we evaluate as species under each given taxonomic framework), until we arrive at very small and narrowly defined species units, which may still include some amount of variability and the ability to hybridize with closely related, narrowly defined species. During the processes of decreasing the degree of disparity of morphological and molecular taxonomic units, the degree of their ‘crypticity’ is at first greatly increased because we are further attempting to find distinguishing features within increasingly smaller taxonomic units.

Therefore, instead of the very problematic division of this multilevel continuum into ‘non- cryptic’ and ‘cryptic’ components, we conclude that almost every species potentially hides numerous smaller and smaller units that need careful detection and description using an ever greater differentiating system of morphological and molecular characters, as was presented here for this case of ‘cryptic’ morphs within a nudibranch species complex (see detailed morphological comparative remarks after description of every species above). The problem was not in the absence of “physically distinguishable morphological characters”, but in the absence of enough finely differentiated units in the taxonomic framework that would have allowed species distinction at a much finer level.

Using the new framework established here for the *T*. *caerulea* species complex (Figs 1–12; Tables S1–S5), which comprises smaller and more differentiated taxonomic levels, we can now start to further explore the taxonomic diversity within that complex, with the additional possibility that we will detect new, even smaller and more finely differentiated morphological and molecular taxonomic levels. So, the possible new taxonomically distinguishing characters of potentially (not yet discovered) smaller levels of taxonomic diversity within *T*. *caerulea* (i.e. not yet discovered potential new species in that complex) would be very ‘cryptic’ and would have inevitably been considered just small variations within the previous framework, but within the new morphological and molecular framework, they will be ‘less cryptic’ than before. This general consideration and the remarkable practical case in the present study clearly show that it is meaningless to use this simplistic ‘cryptic-pseudocryptic’ species pairing while dealing with the enormous continuum of highly differentiated diversity at smaller and smaller levels, each of which is notably more and more ‘cryptic’ than the previous one. Instead, we are proposing two interlinked central implications of the present study: “from ‘cryptic’ to obvious species” within more and more finely differentiated numerous taxonomic levels.

Furthermore, because underestimation of the epigenetic mechanisms in taxonomic practice, the ‘cryptic’ species label may prevent the discovery of such an important evolutionary phenomenon as paedomorphosis, which is now recognized in different groups^23,26,62,63^. While we were analysing integrative morphological and molecular data for this nudibranch species complex we discovered a new Black Sea species, *Trinchesia diljuvia* sp. n., that is externally similar to the Mediterranean *T*. *morrowae* sp. n. (from which it is molecularly considerably diverged) and thus can be classified under the “low morphological disparity” general paradigm^9^ as morphologically uniform, but actually demonstrates stable and clear paedomorphic features (Figs 1, 9 and 12). The formation of the modern Black Sea, approximately 8000–9000 years ago^64^, makes the present example of paedomorphosis-related speciation in the new nudibranch species share the top spot for the most rapid animal speciation in the world. Only one recent comparable study on a marine fish suggested a very rapid degree of speciation of similar age (ca. 6000-8000 years ago)^65^.

Under this fine-scale multilevel species framework, morphological and molecular data, as well the other biological data, should equally contribute to the definition and description of any species. Most recent data show that taxonomists are gradually moving in this direction. Among 55 species of the putative ‘cryptic’ species of the amphipod crustacean which were recently investigated by integrative methods, biological differences (other than molecular differences) were not yet found for only four species^5^. A study on a polychaete family recently appeared which does not refute the possibility of finding morphologically distinguishing characters even within an apparent ‘mega-cryptic’ species complex^66^.

### Concluding remarks on the ‘cryptic’ species problem

How to name morphologically difficult-to-distinguish species diversity is not just a terminological problem^9,10^ but a serious theoretical and practical problem. Initially, the term ‘cryptic species’ has been applied when considerable molecular divergence was discovered within apparently morphologically similar species groups^3,18,67^. However, currently the term ‘cryptic species’ is greatly over used because researchers tend to firstly investigate molecular data and only secondly investigate morphology. Thus, for such studies all species are ‘cryptic’ before molecular study and afterwards they are named ‘pseudocryptic’ species^18,52^. Despite that, in our present study we show that previously recognized morphologically cryptic morphs in a nudibranch species complex are concordant with the newly obtained molecular differences and constitute well-defined morphological and molecular taxonomic units, and this species complex can therefore be termed simultaneously ‘cryptic’ and ‘non-cryptic’. Thus, the degree of ‘crypticity’ is a subjective and uncertain measure^68^.

We therefore propose to avoid the terms ‘cryptic’/’pseudocryptic’ species and in cases where taxonomy is not yet settled use the more neutral terms ‘hidden’ or ‘potentially unraveled/undiscovered’ diversity. The following scheme to explore hidden diversity is suggested: 1) Within a broader taxonomic framework, detect potential cases where species are difficult to distinguish by morphological data; 2) These difficult cases should be studied exhaustively at the current level of technological development; 3) Morphological and molecular methods should be applied in concordance, and ecological and any other relevant data can equally be primary sources to reveal fine but taxonomically reliable differences; 4) As a result of setting the ‘finer and finer taxonomic scale’ in a species complex, a new, more differentiated taxonomic framework will be established. Presumption of the ‘existence of morphological differences’ should be applied at every level of taxonomic study. Justification of such presumption is the biological impossibility of the existence of two genetically different but morphologically completely identical species. Accordingly, if in some cases we are unable to present definite morphological differences this means that we are just currently unable to detect them and need to postpone this unresolved question for a future study, but we cannot just state that morphological differences do not exist.

Facing the rapid extinction of many organisms, including marine ones^69,70^, we should concentrate on describing the patterns of this immense fine-scale multilevel diversity, rather than relying on attempts to merely roughly distinguish ‘cryptic’ species from ‘non-cryptic’ ones. Putative “body doubles”^3,71^ should, under scrutiny, always turn out to be neither doubles nor cryptic, but currently it has become fashionable to name any apparently taxonomically difficult group a ‘cryptic species’. Continued use of this term will only add ambiguity to the immense field of biodiversity, where an enormous number of species still need description, and the ‘cryptic’ concept is rather an obstacle than an aid. The term ‘cryptic species’ should only be regarded as a temporary state. Though the ‘cryptic species concept’ has played a role in emphasizing the importance of reviewing morphologically difficult to distinguish species, currently there is a necessity for a new framework for the species concept itself ^5,72^. The present study thus demands the establishment of such a new, fine-scale multilevel species paradigm through which can we approach a less subjective understanding of what ‘high’ and ‘low’ taxonomic disparity is, and is far from the simplistic ‘cryptic species’ notion which is currently dominant.

## Supplementary information

Supplementary Information

## References

[1] Bickford D (2007). Cryptic species as a window on diversity and conservation. Tren. Ecol. Evol..

[2] Gill BA (2016). Cryptic species diversity reveals biogeographic support for the ‘mountain passes are higher in the tropics’ hypothesis. Proc. R. Soc. Lond. B.

[3] Saéz AG, Lozano E (2005). Body doubles. Nature.

[4] Jörger KM, Schrödl M (2013). How to describe a cryptic species? Practical challenges of molecular taxonomy. Front. Zool..

[5] Fišer C, Robinson CT, Malard F (2018). Cryptic species as a window into the paradigm shift of the species concept. Mol. Ecol..

[6] Knowlton N (1993). Sibling species in the sea. Ann. Rev. Ecol. Syst..

[7] Franks DW, Noble J (2004). Warning signals and predator–prey coevolution. Proc. R. Soc. Lond. B.

[8] Korshunova TA, Martynov AV, Bakken T, Picton BE (2017). External diversity is restrained by internal conservatism: New nudibranch mollusc contributes to the cryptic species problem. Zool. Scripta.

[9] Struck TH (2018). Finding evolutionary processes hidden in cryptic species. Trends Ecol. Evol..

[10] Heethoff M (2018). Cryptic species – conceptual or terminological chaos?. Trends Ecol. Evol..

[11] Beermann J (2018). Cryptic species in a well-known habitat: applying taxonomics to the amphipod genus *Epimeria* (Crustacea, Peracarida). Sci. Rep..

[12] Kanturski M, Lee Y, Choi J, Lee S (2018). DNA barcoding and a precise morphological comparison revealed a cryptic species in the *Nippolachnus piri* complex (Hemiptera: Aphididae: Lachninae). Sci. Rep..

[13] Okanishi M, Sentoku A, Martynov A, Fujita T (2018). A new cryptic species of *Asteronyx* Müller and Troschel, 1842 (Echinodermata: Ophiuroidea), based on molecular phylogeny and morphology, from off Pacific coast of Japan. Zool. Anz..

[14] Garnett ST, Christidis L (2017). Taxonomy anarchy hampers conservation. Nature.

[15] Thomson SA (2018). Taxonomy based on science is necessary for global conservation. PLoS Biol..

[16] Thompson, T. E. & Brown, G. H. *Biology of opisthobranch molluscs*. *Vol*. *2*. (The Ray Society Publications, 1984).

[17] Anderson, J. & Picton, B. *Scottish Nudibranchs*. (Kindle Edition, 2017).

[18] Sáez AG (2003). Pseudo-cryptic speciation in coccolithophores. PNAS.

[19] Lindsay T, Valdés Á (2016). The model organism *Hermissenda crassicornis* (Gastropoda: Heterobranchia) is a species complex. PLoS ONE.

[20] Zhang F (2018). Species delimitation in the morphologically conserved *Coecobrya* (Collembola: Entomobryidae): A case study integrating morphology and molecular traits to advance current taxonomy. Zool. Scripta.

[21] Karanovic T, Djurakic M, Eberhard SM (2016). Cryptic species or inadequate taxonomy? Implementation of 2D geometric morphometrics based on integumental organs as landmarks for delimitation and description of copepod taxa. Syst. Biol..

[22] Furfaro, G., Picton, B., Martynov, A. & Mariottini, P. *Diaphorodoris alba* Portmann & Sandmeier, 1960 is a valid species: molecular and morphological comparison with *D*. *luteocincta* (M. Sars, 1870) (Gastropoda: Nudibranchia). *Zootaxa*, **4193**, 304–316 (2016). 10.11646/zootaxa.4193.2.6 27988719

[23] Korshunova TA, Lundin K, Malmberg K, Picton B, Martynov AV (2018). First true brackish water nudibranch mollusc provides new insights for phylogeny and biogeography and reveals paedomorphosis driven evolution. PLoS ONE.

[24] Morrow CC (2012). Congruence between nuclear and mitochondrial genes in Demospongiae: A new hypothesis for relationships within the G4 clade (Porifera: Demospongiae). Mol. Phyl. Evol..

[25] Korshunova TA (2017). Polyphyly of the traditional family Flabellinidae affects a major group of Nudibranchia: aeolidacean taxonomic reassessment with descriptions of several new families, genera, and species (Mollusca, Gastropoda). ZooKeys.

[26] Korshunova TA, Martynov AV, Picton BE (2017). Ontogeny as an important part of integrative taxonomy in tergipedid aeolidaceans (Gastropoda: Nudibranchia) with a description of a new genus and species from the Barents Sea. Zootaxa.

[27] Korshunova TA, Fletcher K, Lundin K, Picton BE, Martynov AV (2018). The genus *Zelentia* is an amphi-boreal taxon expanded to include three new species from the North Pacific and Atlantic oceans (Gastropoda: Nudibranchia: Trinchesiidae). Zootaxa.

[28] Puillandre N, Lambert A, Brouillet S, Achaz G (2011). ABGD, Automatic Barcode Gap Discovery for primary species delimitation. Mol. Ecol..

[29] Struck (2018). Cryptic species – more than terminological chaos: A reply to Heethoff. Trends Ecol. Evol..

[30] Dayrat B (2005). Toward integrative taxonomy. Biol. J. Linn. Soc..

[31] Picton, B.E. & Morrow, C. *A field guide to the nudibranchs of the British Isles*. (Immel Publishing, 1994).

[32] Padula V, Araújo AK, Matthews-Cascon H, Schrödl M (2014). Is the Mediterranean nudibranch *Cratena peregrina* present in the Brazilian coast? Integrative species delimitation and description of *Cratena minor* n. sp. J. Moll. Stud..

[33] Hayward, P. J. & Ryland, J. S. *Handbook of the marine fauna of north-west Europe*, 2nd ed. (Oxford University Press 2017).

[34] Schmekel L, Portmann A (1982). Opisthobranchia des Mittelmeeres, Nudibranchia und Saccoglossa. Fauna e flora del Golfo di Napoli.

[35] Montagu G (1804). Description of several marine animals found on the south coast of Devonshire. Trans. Linn. Soc. London.

[36] Verany, G. B. Catalogo degli animali invertebrati marini del Golfo di Genova e Nizza. (Genova: Tipografia Ferrando 1846).

[37] Dalyell, J. G. Observations on life amidst the various forms of the humbler tribes of animated nature: with practical comments and illustrations. (London: John van Voorst 1853).

[38] Alder J, Hancock A (1846). Notices of some new and rare British species of naked Mollusca. Ann. Mag. Nat. Hist..

[39] Herdman WA (1881). Additional notes to the invertebrate fauna of Lamlash Bay. Proc. Roy. Phys. Soc. Edin..

[40] Miller MC (1961). Distribution and food of the nudibranchiate Mollusca of the south of the Isle of Man. J Anim. Ecol..

[41] Vicente N (1963). Mollusques opisthobranches récoltés en plongée dans le Golfe de Marseille. Rec. Trav. Sta. Mar. Endoume.

[42] Fraïsse C, Belkhir K, Welch JJ, Bierne N (2016). Local interspecies introgression is the main cause of extreme levels of intraspecific differentiation in mussels. Mol. Ecol..

[43] Crossman CA, Taylor EB, Barrett-Lennard LG (2016). Hybridization in the Cetacea: widespread occurrence and associated morphological, behavioral, and ecological factors. Ecol. Evol..

[44] Struck TH, Koczula J, Stateczny D, Meyer C, Purschke G (2017). Two new species in the annelid genus *Stygocapitella* (Orbiniida, Parergodrilidae) with comments on their biogeography. Zootaxa.

[45] Fraga MF (2005). Epigenetic differences arise during the lifetime of monozygotic twins. PNAS.

[46] Wong AHC, Irving I, Petronis GA (2005). Phenotypic differences in genetically identical organisms: the epigenetic perspective. Human Mol. Gen..

[47] Casadesús J, Low DA (2013). Programmed heterogeneity: epigenetic mechanisms in Bacteria. J. Biol. Chem..

[48] Perez MF, Francesconi M, Hidalgo-Carcedo C, Lehner B (2017). Maternal age generates phenotypic variation in *Caenorhabditis elegans*. Nature.

[49] Singhal Sonal, Hoskin Conrad J, Couper Patrick, Potter Sally, Moritz Craig (2018). A Framework for Resolving Cryptic Species: A Case Study from the Lizards of the Australian Wet Tropics. Systematic Biology.

[50] Wada S, Kameda Y, Chiba S (2013). Long-term stasis and short-term divergence in the phenotypes of microsnails on oceanic islands. Mol. Ecol..

[51] Nygren A (2014). Cryptic polychaete diversity: a review. Zool. Scripta.

[52] Matsuda SB, Gosliner TM (2018). Glossing over cryptic species: Descriptions of four new species of *Glossodoris* and three new species of *Doriprismatica* (Nudibranchia: Chromodorididae). Zootaxa.

[53] Uribe R, Sepúlveda F, Goddard JHR, Valdés Á (2017). Integrative systematics of the genus *Limacia* O. F. Müller, 1781 (Gastropoda, Heterobranchia, Nudibranchia, Polyceridae) in the Eastern Pacific. Mar. Biod., early online view.

[54] Caballer M, Almón B, Pérez J (2016). The sea slug genus *Limacia* Müller, 1781 (Mollusca: Gastropoda: Heterobranchia) in Europe. Cahiers Biol. Mar..

[55] de Queiroz K (2007). Species concepts and species delimitation. Syst. Biol..

[56] Wilson NG, Schrödl M, Halanych K (2009). Ocean barriers and glaciation: evidence for explosive radiation of mitochondrial lineages in the Antarctic sea slug *Doris kerguelenensis* (Mollusca, Nudibranchia). Mol. Ecol.

[57] Valdés Á, Medrano S, Bhave V (2016). A new species of *Cuthona* Alder and Hancock, 1855 (Gastropoda: Heterobranchia: Nudibranchia: Tergipedidae) from the Caribbean Sea. Natitulus.

[58] Padula V (2016). A test of colorbased taxonomy in nudibranchs: molecular phylogeny and species delimitation of the *Felimida clenchi* (Mollusca: Chromodorididae) species complex. Mol. Phyl. Evol..

[59] Mayer F, von Helversen O (2001). Cryptic diversity in European bats. Proc. R. Soc. Lond. B.

[60] Wiemers M, Fiedler K (2007). Does the DNA barcoding gap exist? – a case study in blue butterflies. Front. Zool..

[61] Takeuchi N, Kaneko K, Hogeweg P (2016). Evolutionarily stable disequilibrium: Endless dynamics of evolution in a stationary population. Proc. R. Soc. Lond. B.

[62] Bhullar BA (2012). Birds have paedomorphic dinosaur skulls. Nature.

[63] Bocak L, Kundata R, Fernandez CA, Vogler AP (2016). The discovery of Iberobaeniidae (Coleoptera: Elateroidea): a new family of beetles from Spain, with immatures detected by environmental DNA sequencing. Proc. R. Soc. Lond. B.

[64] Herrle JO (2018). Black Sea outflow response to Holocene meltwater events. Sci. Rep..

[65] Momigliano P (2017). Extraordinarily rapid speciation in a marine fish. PNAS.

[66] Nygren (2018). A mega-cryptic species complex hidden among one of the most common annelids in the North East Atlantic. PLoS ONE.

[67] Paris CA, Wagner FS, Wagner WH (1989). Cryptic species, species delimitation, and taxonomic practice in the homosporous ferns. Amer. Fern J..

[68] Lajus D, Sukhikh N, Alekseev V (2015). Cryptic or pseudocryptic: can morphological methods inform copepod taxonomy? An analysis of publications and a case study of the *Eurytemora affinis* species complex. Ecol Evol..

[69] Ripple WJ (2017). World scientists’ warning to humanity: a second notice. BioScience.

[70] Schrödl, M. & Häussermann, V. BiodiversiTOT. Die globale Artenvielfalt jetzt entdecken, erforschen und erhalten. (Norderstedt, 2017).

[71] Lee MS, Oliver PM (2016). Count cryptic species in biodiversity tally. Nature.

[72] Zachos, F. E. Species concepts in biology: historical development, theoretical foundations and practical relevance. (Springer Nature, 2016).

