# Supplementary material for: Multilevel fine-scale diversity challenges the ‘cryptic species’ concept
Source: Sci Rep. 2019 May 1;9:6732. doi: 10.1038/s41598-019-42297-5 (PMC6494890; doi:10.1038/s41598-019-42297-5)
Supplement: Supplementary file 1 — Supplementary Information [file 41598_2019_42297_MOESM1_ESM.pdf]

# Supplementary Information
# for the article Multilevel fine-scale diversity challenges the 'cryptic species' concept

Tatiana Korshunova[1, 2], Bernard Picton[3,4], Giulia Furfaro[5], Paolo Mariottini[5], Miquel Pontes[6], Jakov Prkić[7], Karin Fletcher[8], Klas Malmberg[9], Kennet Lundin[10,11], Alexander Martynov[2]

[1]Koltzov Institute of Developmental Biology RAS, 26 Vavilova Str., 119334 Moscow, Russia. [2]Zoological Museum, Moscow State University, Bolshaya Nikitskaya Str. 6, 125009 Moscow, Russia. [3]National Museums Northern Ireland, Holywood, Northern Ireland, BT18 0EU, United Kingdom. [4]Queen's University, Belfast, Northern Ireland, United Kingdom. [5]Department of Science, University of "Roma Tre", Viale G. Marconi 446, I-00146 Rome, Italy. [6] Vida Marina VIMAR, Barcelona, Spain. [7]Getaldiceva 11, C 21000 Split, Croatia. [8]Port Orchard, Washington, 98366, USA. [9]Aquatilis, Nostravägen 11, S-41743, Gothenburg, Sweden. [10]Gothenburg Natural History Museum, Box 7283, SE-40235, Gothenburg, Sweden. [11]Gothenburg Global Biodiversity Centre, Box 461, SE-40530, Gothenburg, Sweden. Correspondence and requests for materials should be addressed to AM (martynov@zmmu.msu.ru)

## Text (Materials and Methods)

## Sample data

Material for this study was obtained by scuba diving at widely separated locations in Europe. Specimens were collected in Norway (west coast, near the mouth of the Sogne fjord, Gulen Dive Resort), Sweden (west coast, Bohuslän county, Smögen Dive Resort), Ireland (Killary Harbour), United Kingdom (Northern Ireland, Strangford Lough), Spain (L'Estartit, Girona), Italy, France, Croatia and in the Black Sea, Russia (Yalta region). The specimens were preserved in 95–96% or 70% ethanol and deposited in the Zoological Museum of Lomonosov Moscow State University (ZMMU), in the Gothenburg Natural History Museum (GNM) and National Museums Northern Ireland (BELUM.Mn), and Department of Science of the Roma Tre University (RM3).

## Morphological analysis

The morphology of the nudibranchs and their egg masses was studied under a stereomicroscope and using Nikon D810 and Nikon D600 digital cameras. For the description of internal features both preserved and fresh specimens (when available) were dissected under the stereomicroscope. The buccal mass of each specimen was extracted and processed in 10% sodium hypochlorite solution. The features of the jaws of each species were analysed under the stereomicroscope and scanning electron microscope and were then drawn. The coated radulae were examined and photographed using a scanning electron microscope (CamScan, JSM). The reproductive systems were also examined and drawn using the stereomicroscope.

## Molecular analysis

A total of 17 specimens were successfully sequenced for the mitochondrial genes cytochrome c oxidase subunit I (COI), 16S rRNA, and the nuclear genes Histone 3 (H3) (see Table for the primers). Of these, one specimen was sequenced in the United Kingdom, three specimens were sequenced in Rome, the others in Moscow. Additional molecular data for 12 specimens of nudibranchs were obtained from GenBank (see Table S1). DNA extraction procedure, PCR amplification options, and sequence obtainment is previously described in detail (Korshunova, T.A. *et al.* 2017a, 2017b,2018; Morrow *et al.* 2012, Furfaro 2016 *et al.*). Protein coding sequences were translated into amino acids for confirmation of the alignment. All sequences were deposited in GenBank (see Supporting Information, Table S1, highlighted in bold). Original data and publicly available sequences were aligned with the MAFFT algorithm (Katoh *et al.*2002). Separate analyses were conducted for COI (658 bp), 16S (442 bp), H3 (328 bp) and concatenated data (1428 bp). Evolutionary models for each data set were selected using MrModelTest 2.3 under the Akaike information criterion (Nylander et al. 2004 ,Akaike 1974). Two different phylogenetic methods, Bayesian inference (BI) and Maximum Likelihood (ML), were used to infer evolutionary relationships. Bayesian estimation of posterior probability was performed in MrBayes 3.2 (Ronquist et al. 2012). Four Markov chains were sampled at intervals of 500 generations. Analysis was started with random starting trees and $10^7$ generations. ML analysis was performed using RAxML 7.2.8 (Stamatakis et al. 2008) with 1000 bootstrap replicates. Final phylogenetic tree images were rendered in FigTree 1.4.2. To evaluate the genetic distribution of the different haplotypes, a haplotype network for the COI molecular data was reconstructed using Population Analysis with Reticulate Trees (PopART, http://popart.otago.ac.nz) with the TCS network method. The program Mega7 (Kumar et al. 2016) was used to calculate the minimum uncorrected *p*-distances between all the sequences. Intra- and intergroup genetic distances were also examined. Additionally, Automatic Barcode Gap Discovery (ABGD) was used to define species (Puillandre et al. 2011). Alignment from the COI marker for *Trinchesia* specimens were submitted and processed in ABGD using the Jukes-Cantor (JC69) and Kimura (K80) models and the following settings: a prior for the maximum value of intraspecific divergence between 0.001 and 0.1, 20 recursive steps.

## Primers used for amplification.

| Name | 5′→3′ | References |
|---|---|---|
| LCO 1490 | GGTCAACAAATCATAAAGATATTGG | Folmer *et al.*, 1994 |
| HCO 2198 | TAAACTTCAGGGTGACCAAAAAATCA | Folmer *et al.*, 1994 |
| 16S arL | CGCCTGTTTAACAAAAACAT | Palumbi *et al.* 2002 |
| 16S R | CCGRTYTGAACTCAGCTCACG | Puslednik & Serb, 2008 |
| H3 AF | ATGGCTCGTACCAAGCAGACGG | Colgan *et al.*, 1998 |
| H3 AR | ATATCCTTGGGCATGATGGTGAC | Colgan *et al.*, 1998 |

## References


Akaike, H. A new look at the statistical model identification. *IEEE Trans Automat Control*, **19,** 716–723 (1974).

Colgan, DJ, McLauchlan, A, Wilson, GDF, Livingston, SP, Edgecombe, GD, Macaranas, J,

Cassis, G & Gray, MR Histone H3 and U2 snRNA DNA sequences and arthropod mo-lecular evolution. *Australian Journal of Zoology*, **46**, 419–437 (1998).

Folmer O, Black M, Hoeh W, Lutz R, Vrijenhoek R. DNA primers for amplification of mitochondrial cytochrome c oxidase subunit I from diverse metazoan invertebrates. Molecular Mar Biol Biotech., **3**, 294-299 (1994).

Furfaro, G, Picton, B, Martynov, A & Mariottini, P *Diaphorodoris alba* Portmann & Sandmeier, 1960 is a valid species: molecular and morphological comparison with *D. luteocincta* (M. Sars, 1870) (Gastropoda: Nudibranchia). *Zootaxa*, **4193,** 304–316 (2016).

Katoh, K, Misawa, K., Kuma, K, Miyata, T. MAFFT: a novel method for rapid multiple sequence alignment based on fast Fourier transform, *Nucleic Acids Res.* **30,** 3059–3066 (2002).

Korshunova, TA *et al.* Polyphyly of the traditional family Flabellinidae affects a major group of Nudibranchia: aeolidacean taxonomic reassessment with descriptions of several new families, genera, and species (Mollusca, Gastropoda). *ZooKeys* **717,** 1–139 (2017a).

Korshunova, TA, Lundin, K, Malmberg, K, Picton, B & Martynov, AV First true brackish water nudibranch mollusc provides new insights for phylogeny and biogeography and reveals paedomorphosis driven evolution. *PLoS ONE* **13,** e0192177; 10.1371/journal.pone.0192177 (2018).

Korshunova, TA, Martynov, AV, Bakken, T & Picton, BE External diversity is restrained by internal conservatism: New nudibranch mollusc contributes to the cryptic species problem. *Zool. Scripta* **46,** 683–692 (2017b).

Kumar, S, Stecher, G, Tamura, K. MEGA7: Molecular evolutionary genetics analysis version 7.0 for bigger datasets. *Mol. Biol. Evol.* **33,** 1870–1874 (2016).

Morrow, CC, Picton, BE, Erpenbeck, D, Boury-Esnault, N, Maggs, CA & Allcock, A L Congruence between nuclear and mitochondrial genes in Demospongiae: A new hypothesis for relationships within the G4 clade (Porifera: Demospongiae). *Mol. Phyl. Evol.* **62,** 174–190 (2012).

Nylander, JA, Ronquist, F, Huelsenbeck, JP, Nieves-Aldrey, JL Bayesian phylogenetic analysis of combined data. *Syst. Biol.* **53,** 47–67 (2004).

Palumbi SR, Martin AP, Romano S, McMillan WO, Stice L, Grabowski G. The simple fool's guide to PCR. Honolulu: University of Hawaii, (2002).

Puillandre, N, Lambert, A, Brouillet, S, & Achaz, G. ABGD, Automatic Barcode Gap Discovery for primary species delimitation. Molecular Ecology, **21**, 1864–1877 (2011).

Puslednik L, Serb JM. Molecular phylogenetics of the Pectinidae (Mollusca: Bivalvia) and effect of increased taxon sampling and outgroup selection on tree topology. Mol Phyl Evol. **48**, 1178-1188 (2008).

Ronquist, F *et al.* MrBayes 3.2: Efficient Bayesian phylogenetic inference and model choice across a large model space. *Syst. Biol.* **61,** 539–542 (2012).

Stamatakis, A, Hoover, P, Rougemont, J A Rapid bootstrap algorithm for the RAxML web-servers. *Syst. Biol.* **75**, 758–771 (2008).

## Tables S1–S5

### Table S1. List of specimens used for molecular analyses.

| Species | Locality | Registration | GenBank accession nos. | | |
|---|---|---|---|---|---|
| | | | COI | 16S | H3 |
| *Diaphoreolis lagunae* (O'Donoghue, 1926) | California | CAS179465a | KY128956 | KY128749 | KY128543 |
| *Diaphoreolis viridis* (Forbes, 1840) | Russia, White Sea | ZMMU:Op-537 | MG266028 | MG266026 | MG266029 |
| *Trinchesia diljuvia* **sp.n.** | Russia, Black Sea | ZMMU:Op-642 | **MK587917** | **MK587933** | **MK587903** |
| *Trinchesia diljuvia* **sp.n.** | Russia, Black Sea | ZMMU:Op-643 | **MK587918** | **MK587934** | **MK587904** |
| *Trinchesia caerulea* (Montagu, 1804) | Norway, Gulen | ZMMU:Op-622 | MG266024 | MG266022 | MG266025 |
| *Trinchesia caerulea* (Montagu, 1804) | North Sea | - | AF249807 | - | - |
| *Trinchesia caerulea* (Montagu, 1804) | Ireland | ZMMU:Op-646 | **MK587928** | **MK587930** | - |
| *Trinchesia caerulea* (Montagu, 1804) | Spain, L'Estartit, Girona | ZMMU:Op-648 | **MK587915** | **MK587931** | **MK587898** |
| *Trinchesia caerulea* (Montagu, 1804) | Spain, L'Estartit, Girona | ZMMU:Op-649 | **MK587916** | **MK587932** | **MK587899** |
| *Trinchesia caerulea* (Montagu, 1804) | Italy, Tor Paterno | RM3_309 | **MK587912** | - | **MK587896** |
| *Trinchesia caerulea* (Montagu, 1804) | Italy, Tor Paterno | RM3_333 | **MK587913** | **MK587929** | **MK587897** |
| *Trinchesia caerulea* (Montagu, 1804) | Croatia, Ugljan Island | ZMMU Op-647 | **MK587914** | - | - |
| *Trinchesia cuanensis* **sp.n.** | United Kingdom | GNM 9099 | KY128911 | KY128703 | KY128498 |
| *Trinchesia cuanensis* **sp.n.** | United Kingdom | ZMMU: Op-650 | **MK587922** | MK587936 | MK587906 |
| *Trinchesia cuanensis* **sp.n.** | United Kingdom | GNM 9054 | **MK587920** | **MK587935** | **MK587905** |
| *Trinchesia cuanensis* **sp.n.** | United Kingdom | BELUM.Mn.2018.2 | **MK587919** | **-** | **-** |
| *Trinchesia cuanensis* **sp.n.** | North Sea, Sweden | GNM 9243 | **MK587921** | **-** | **-** |
| *Trinchesia lenkae* Martynov, 2002 | Japan: Hokkaido | ZMMU:Op-619 | MG242334 | MG242333 | **MK587900** |
| *Trinchesia lenkae* Martynov, 2002 | Japan: Hokkaido | ZMMU:Op-620 | MG242336 | MG242335 | **MK587901** |
| *Trinchesia lenkae* Martynov, 2002 | Russia: Sea of Japan | ZMMU:Op-621 | MG242338 | MG242337 | **MK587902** |
| *Trinchesia morrowae* **sp.n.** | France, Banyuls-sur-Mer | ZMMU:Op-535 | **MK587923** | **MK587937** | **MK587907** |
| *Trinchesia morrowae* **sp.n.** | France, Banyuls-sur-Mer | ZMMU:Op-653 | **MK587926** | **MK587940** | **MK587910** |

| | | | | | |
|---|---|---|---|---|---|
| *Trinchesia morrowae* **sp.n.** | France, Banyuls-sur-Mer | ZMMU Op-656 | **MK587927** | **MK587941** | **MK587911** |
| *Trinchesia morrowae* **sp.n.** | Spain, L'Estartit, Girona | ZMMU:Op-651 | **MK587924** | **MK587938** | **MK587908** |
| *Trinchesia morrowae* **sp.n.** | Spain, L'Estartit, Girona | ZMMU Op-652 | **MK587925** | **MK587939** | **MK587909** |
| *Trinchesia morrowae* **sp.n.** | Spain | CAS185199 | KY128922 | KY128717 | KY128510 |
| *Trinchesia ornata* (Baba, 1937) | Hawaii | CAS180344 | KY128967 | KY128758 | KY128553 |
| *Trinchesia sibogae* (Bergh, 1905) | Philippines | CAS177489 | KY128975 | KY128767 | KY128562 |
| *Trinchesia speciosa* (Macnae, 1954) | South Africa | CAS176913 | KY128996 | KY128788 | KY128583 |
| *Trinchesia speciosa* (Macnae, 1954) | South Africa | CAS176954 | KY128998 | KY128790 | KY128585 |

## Table S2. Maximum intragroup (highlighted in bold) and minimum intergroup genetic distances (%) for COI marker in *Trinchesia* species.

| | *T. caerulea* | *T. cuanensis* **sp.n.** | *T. morrowae* **sp.n.** | *T. diljuvia* **sp.n.** | *T. lenkae* | *T. speciosa* | *T. ornata* | *T. sibogae* |
|---|---|---|---|---|---|---|---|---|
| *T.caerulea* | **1.25** | 7.20 | 10.95 | 11.89 | 16.74 | 15.18 | 17.45 | 17.06 |
| *T. cuanensis* **sp.n.** | 7.20 | **1.88** | 11.27 | 12.99 | 18.5 | 16.43 | 17.45 | 17.53 |
| *T. morrowae* **sp.n.** | 10.95 | 11.27 | **2.18** | 2.98 | 17.84 | 14.71 | 15.88 | 16.12 |
| *T. diljuvia* **sp.n.** | 11.89 | 12.99 | 2.98 | **0.16** | 17.37 | 15.96 | 16.67 | 17.53 |
| *T. lenkae* | 16.74 | 18.5 | 17.84 | 17.37 | **0.31** | 16.12 | 13.36 | 18.94 |
| *T. speciosa* | 15.18 | 16.43 | 14.71 | 15.96 | 16.12 | **0.78** | 14.62 | 16.28 |
| *T. ornata* | 17.45 | 17.45 | 15.88 | 16.67 | 13.36 | 14.62 | **-** | 17.14 |
| *T. sibogae* | 17.06 | 17.53 | 16.12 | 17.53 | 18.94 | 16.28 | 17.14 | **-** |

## Table S3. Maximum intragroup (highlighted in bold) and minimum intergroup genetic distances (%) for 16S marker in *Trinchesia* species.

| | *T. caerulea* | *T. cuanensis* **sp.n.** | *T. morrowae* **sp.n.** | *T. diljuvia* **sp.n.** | *T. lenkae* | *T. speciosa* | *T. ornata* | *T. sibogae* |
|---|---|---|---|---|---|---|---|---|
| *T.caerulea* | **0.25** | 1.39 | 5.31 | 6.47 | 11.52 | 11.52 | 10.54 | 11.03 |
| *T. cuanensis* **sp.n.** | 1.39 | **0.46** | 6.0 | 6.24 | 12.01 | 11.55 | 11.32 | 10.62 |
| *T. morrowae* **sp.n.** | 5.31 | 6.0 | **0.23** | 1.62 | 11.32 | 10.39 | 11.55 | 10.39 |
| *T. diljuvia* **sp.n.** | 6.47 | 6.24 | 1.62 | **0.46** | 11.55 | 10.39 | 11.78 | 10.62 |
| *T. lenkae* | 11.52 | 12.01 | 11.32 | 11.55 | **0.46** | 8.78 | 4.39 | 10.39 |
| *T. speciosa* | 11.52 | 11.55 | 10.39 | 10.39 | 8.78 | **0** | 8.78 | 12.01 |
| *T. ornata* | 10.54 | 11.32 | 11.55 | 11.78 | 4.39 | 8.78 | **-** | 11.09 |
| *T. sibogae* | 11.03 | 10.62 | 10.39 | 10.62 | 10.39 | 12.01 | 11.09 | **-** |

## Table S4. Maximum intragroup (highlighted in bold) and minimum intergroup genetic distances (%) for H3 marker in *Trinchesia* species.

| | *T. caerulea* | *T. cuanensis* **sp.n.** | *T. morrowae* **sp.n.** | *T. diljuvia* **sp.n.** | *T. lenkae* | *T. speciosa* | *T. ornata* | *T. sibogae* |
|---|---|---|---|---|---|---|---|---|
| *T.caerulea* | **0.66** | 0.98 | 1.63 | 1.63 | 4.25 | 5.88 | 4.9 | 3.27 |
| *T. cuanensis* **sp.n.** | 0.98 | **0** | 1.63 | 1.31 | 4.58 | 6.21 | 5.23 | 3.59 |
| *T. morrowae* **sp.n.** | 1.63 | 1.63 | **0.65** | 0.98 | 4.58 | 6.21 | 5.23 | 4.58 |
| *T. diljuvia* **sp.n.** | 1.63 | 1.31 | 0.98 | **0** | 4.58 | 6.21 | 5.23 | 4.25 |
| *T. lenkae* | 4.25 | 4.58 | 4.58 | 4.58 | **0** | 2.61 | 2.29 | 4.90 |
| *T. speciosa* | 5.88 | 6.21 | 6.21 | 6.21 | 2.61 | **0** | 3.59 | 7.52 |
| *T. ornata* | 4.9 | 5.23 | 5.23 | 5.23 | 2.29 | 3.59 | **-** | 5.56 |
| *T. sibogae* | 3.27 | 3.59 | 4.58 | 4.25 | 4.90 | 7.52 | 5.56 | **-** |

**Table S5. Morphological characters that allow species of the *Trinchesia caerulea* complex to be distinguished.**

| | **Adult length (live, max)** | **Anterior ceratal rows** | **Anterior foot structures** | **White line across dorsum** | **Colour zones of cerata** | **Colour of digestive gland in ceratal bases** | **Lower orange band on cerata** | **Upper narrow black band on cerata** | **Radula** | **Receptaculum seminis** | **Stylet of the copulative organ** |
|---|---|---|---|---|---|---|---|---|---|---|---|
| *T. caerulea* | 21 mm | Commonly 4 | Long foot corners | Absent | Present | Greenish to light grayish | Weak or reduced | Absent | 57-63 x 0.1.0 | Oval, large, 8-shaped, but without lateral additional chamber | Short |
| *T. cuanensis* **sp.n.** | 15 mm | Commonly 4 | Short foot corners | Absent | Present | Blackish to dark grayish | Weak or reduced | Present | 64 x 0.1.0 | Rounded, large, with long convoluted additional chamber | Very long |
| *T. morrowae* **sp.n.** | 10 mm | Commonly 3, rarely 4 | Angular projections | Present | Present | Grayish to yellowish | Distinct, often broad | Absent | 55-57x 0.1.0 | Oval, small without lateral additional chamber | Short |
| *T. diljuvia* **sp.n.** | 4.5 mm | Commonly 2, rarely 3 | Distinct projections absent | Present | Absent | No distinct colour zones, dark grayish to light brownish or light greenish and covered with white or sometimes with light bluish dispersed pigment | Absent | Absent | 27–34 x 0.1.0 | Elongated, without lateral additional chamber | Relatively short |
